# Risk-based stratified primary care for common musculoskeletal pain presentations (STarT MSK): a cluster-randomised, controlled trial

**DOI:** 10.1016/S2665-9913(22)00159-X

**Published:** 2022-07-15

**Authors:** Jonathan C Hill, Stefannie Garvin, Kieran Bromley, Benjamin Saunders, Jesse Kigozi, Vince Cooper, Martyn Lewis, Joanne Protheroe, Simon Wathall, Adrian Chudyk, Kate M Dunn, Hollie Birkinshaw, Sue Jowett, Elaine M Hay, Danielle van der Windt, Christian Mallen, Nadine E Foster

**Affiliations:** a Primary Care Centre Versus Arthritis, School of Medicine, Keele University, Staffordshire, UK; b Keele Clinical Trials Unit, Keele University, Staffordshire, UK; c Health Economics Unit, Institute of Applied Health Research, University of Birmingham, Birmingham, UK; d STARS Education and Research Alliance, Surgical Treatment and Rehabilitation Service, University of Queensland and Metro North Health, QLD, Australia

## Abstract

**Background:**

Risk-based stratified care shows clinical effectiveness and cost-effectiveness versus usual primary care for non-specific low back pain but is untested for other common musculoskeletal disorders. We aimed to test the clinical effectiveness and cost-effectiveness of point-of-care risk stratification (using Keele's STarT MSK Tool and risk-matched treatments) versus usual care for the five most common musculoskeletal presentations (back, neck, knee, shoulder, and multi-site pain).

**Methods:**

In this cluster-randomised, controlled trial in UK primary care with embedded qualitative and health economic studies we recruited patients from 24 general practices in the West Midlands region of England. Eligible patients were those aged 18 years or older whose general practitioner (GP) confirmed a consultation for a musculoskeletal presentation. General practices that consented to participate via a representative of the cluster were randomly assigned (1:1) to intervention or usual care, using stratified block randomisation. Researchers involved in data collection, outcome data entry, and statistical analysis were masked at both the cluster and individual participant level. Participating patients were told the study was examining GP treatment of common aches and pains and were not aware they were in a randomised trial. GPs in practices allocated to the intervention group were supported to deliver risk-based stratified care using a bespoke computer-based template, including the risk-stratification tool, and risk-matched treatment options for patients at low, medium, or high risk of poor disability or pain outcomes. There were 15 risk-matched treatment options. In the usual care group, patients with musculoskeletal pain consulting their GP received treatment as usual, typically including advice and education, medication, referral for investigations or tests, or referral to other services. The primary outcome was time-averaged pain intensity over 6 months. All analyses were done by intention to treat. The trial is registered with ISRCTN, ISRCTN15366334.

**Results:**

Between May 1, 2018, and April 30, 2019, 104 GPs from 24 practices (12 per study group) identified 2494 patients with musculoskeletal pain. 1211 (49%) participants consented to questionnaires (534 in the intervention group and 677 in the usual care group), with 1070 (88%) completing the follow-up questionnaire at 6 months. We found no significant difference in time-averaged pain intensity (mean(SD) mean 4·4 [SD 2·3] in the intervention group *vs* 4·6 [2·5] in the control group; adjusted mean difference −0·16, 95% CI −0·65 to 0·34) or in standardised function score (mean −0·06 [SD 0·94] in the intervention group *vs* 0·05 [1·04]; adjusted mean difference −0·07, 95% CI −0·22 to 0·08). No serious adverse events or adverse events were reported. Risk stratification received positive patient and clinician feedback.

**Interpretation:**

Risk stratification for patients in primary care with common musculoskeletal presentations did not lead to significant improvements in pain or function, although some aspects of GP decision making were affected, and GP and patients had positive experiences. The costs of risk-based stratified care were similar to usual care, and such a strategy only offers marginal changes in cost-effectiveness outcomes. The clinical implications from this trial are largely inconclusive.

**Funding:**

National Institute for Health Research.

## Introduction

Musculoskeletal pain, such as back, neck, shoulder, knee, and multi-site pain, is common and burdensome for individuals, health-care providers, and society.^1^ For some people, musculoskeletal problems are short-lived, yet for others painful episodes persist or recur, impacting day-to-day functioning, such as the ability to work, leading to extensive health-care and societal costs.^2^, ^3^

Most patients with musculoskeletal problems are managed in primary care. In the UK, 20% of adults in registered practice populations consult their general practitioner (GP) annually for a musculoskeletal disorder, accounting for one in six GP consultations.^4^ There is a paucity of evidence concerning how to systematically direct patients to optimal treatment in ways that improve patients' outcomes, such as pain and function, and optimise use of health-care resources.^5^, ^6^ The high prevalence of musculoskeletal pain and wide variation in prognosis make it neither feasible nor appropriate to offer intensive or expensive treatments to all individuals.^7^

**Research in context**

**Evidence before this study**

We searched PubMed between Jan 1, 2000, and May 31, 2018 (trial start date), using the search terms “stratified care”, “musculoskeletal pain”, “back pain”, and “osteoarthritis”. The search was restricted to English language publications. We found no previous trials of stratified care for musculoskeletal pain before our study except for our own STarT Back approach for treating non-specific low back pain. Therefore, we used this evidence to refine our STarT Back Tool into a new validated risk stratification tool called the STarT MSK Tool and did a systematic review and consensus studies to determine appropriate risk-matched treatment options for use in primary care, which were tested in this trial.

**Added value of this study**

This trial showed that, although there were some positive changes in clinician decision making, patient-reported outcomes such as pain and function did not improve significantly by using risk-based stratified primary care for patients with the five most common musculoskeletal pain presentations. The costs of stratified care were similar to usual care.

**Implications of all the available evidence**

In contrast to the positive results seen for a stratified care approach for back pain, among patients with musculoskeletal pain in primary care the effectiveness of risk-based stratified care remains inconclusive. Considering the uptake among general practitioners of the stratified intervention was less than 30%, a key future challenge is to find more feasible methods to stratify patients during short consultations, and to provide more effective treatments (such as psychologically informed physiotherapy) for those identified as being at a high risk of persistent disabling pain.

Risk-based stratified care involves targeting treatments according to patients' risk of persistent disabling pain, to maximise treatment benefit and reduce potential harm or unnecessary interventions and cost.^8^ Building on a previously successful primary care model of risk-based stratified care for patients with low back pain in the UK,^9^, ^10^, ^11^ we aimed to test the clinical and cost effectiveness of a similar approach^12^ for patients (at the individual participant level) with the five most common musculoskeletal pain presentations (back, neck, knee, shoulder, or multi-site) compared with usual non-stratified primary care.

## Methods

### Study design and participants

The STarT MSK trial was a parallel-group, pragmatic, cluster-randomised controlled trial with embedded health economic and qualitative studies. A cluster randomised trial was chosen over an individual randomisation design because the intervention involved bespoke electronic medical record templates, which could only be implemented at a practice level without causing a high probability of intervention contamination across study groups. Patients were recruited from 24 general practices in the West Midlands region of England. Practice eligibility criteria were as follows: those using the Egton Medical Information Systems web clinical system (the most commonly used electronic medical record system in the UK), those proficient at using Read codes (diagnostic codes) during musculoskeletal consultations evidenced through an audit of their recent Read coding behaviour, a willingness to undergo the training and support sessions needed to become familiar with the stratified care intervention, a willingness to participate in anonymised aggregated medical record audits of musculoskeletal consultations during the trial recruitment period, and a willingness to engage with the process evaluation. The trial was conducted and analysed according to the published protocol, with no important changes made to the methods after trial commencement.^13^

Potential participants were identified in GP consultations by electronic pop-up computer prompts triggered by appropriate musculoskeletal diagnostic or symptom codes.^14^ An electronic tag was stored in patient medical records when the following criteria were met at the point of consultation by the GP: confirmed eligibility, recorded pain site, recorded patient's pain intensity (0–10 numerical rating scale), and patient verbal consent to participate was recorded. Weekly searches identified electronically tagged medical records. These patients were sent an invitation from their GP to participate in the study (involving providing written informed consent in the baseline questionnaire) and asked to complete monthly questionnaires for 6 months.

Eligible patients were those aged 18 years or older whose GP confirmed a consultation for a musculoskeletal presentation. Those who consented were asked to complete their initial questionnaire within 30 days and give permission for researchers to link survey data with their GP medical record. Patient exclusion criteria were those with indications of serious red flag pathology (eg, recent trauma with clinically significant injury; acute, red, hot swollen joint; suspected fracture; joint infection; cancer; and inflammatory arthropathy, such as rheumatoid arthritis, spondyloarthropathy, polymyalgia rheumatica, and crystal disease [gout]), those with urgent medical care needs (eg, cauda equina syndrome), vulnerable patients (including any patients on the severe and enduring mental health register, those with a diagnosis of dementia, those with a recent diagnosis of a terminal illness, those who had experienced recent trauma or bereavement, or those nearing the end of their life), and those who were unable to communicate in English (both in reading and speaking).

The programme this study is part of received ethics approval from the UK National Health Service (NHS) Research Ethics Committee East Midlands Nottingham 1 (reference 16/EM/0257). All participants provided written informed consent to participate in the research. All methods reported were done in accordance with the relevant guidelines and regulations as outlined in the Declaration of Helsinki.

### Randomisation and masking

General practices that consented to participate via a representative of the cluster were randomly assigned (1:1) to intervention or usual care by the senior statistician (ML) and principal investigator (JCH) by a computer-generated randomisation service from Keele Clinical Trials Unit (with the process overseen by an external statistician), using stratified block randomisation^15^ based on practice patient list size. Masking of individual clinicians was not possible, but researchers involved in data collection, outcome data entry, and statistical analysis were masked at both the cluster and individual participant level, with allocation concealment at the cluster level achieved by each practice having an anonymised code and trial meetings being divided so that masked individuals were not present during discussion about named intervention practices. Participating patients were told the study was examining GP treatment of common aches and pains and were not aware they were in a randomised trial.

### Procedures

An initial pilot phase assessed participant recruitment and follow-up rates over 6 months, trial processes, and adherence to trial protocols. The pilot phase did not achieve the a-priori agreed progression criteria, with lower recruitment and poorer intervention adherence than anticipated, and was changed from a planned internal pilot to an external pilot.^16^, ^17^ Subsequent changes included removing the power to detect differences at the level of each risk stratum to allow a reduction in the main trial's sample size (n=3600 to n=1200), use of a point-of-consultation interview style, rather than self-report style version of the Keele StarT MSK Tool,^12^, ^18^ and revisions to the risk-matched treatment options.^13^ The pilot phase (n=524) did not involve formal analysis of between-group differences in patient outcomes, and these data were not included in the main trial results.

GPs in practices allocated to the intervention group were supported to deliver risk-based stratified care using a bespoke computer-based template, including the risk-stratification tool, and risk-matched treatment options for patients at low, medium, or high risk of poor disability or pain outcomes.^19^, ^20^ Briefly, there were 15 risk-matched treatment options (appendix pp 8–9). The intervention aimed to encourage GPs to use less prescribing of long-term opioids, neuromodulators, muscle relaxants and corticosteroid injections, less unnecessary referrals (eg, to imaging and specialist orthopaedics), and less sick certification (particularly for low-risk patients); and more written self-management advice, simple over-the-counter analgesics, earlier referral to physiotherapy (for patients at medium or high risk), plus further GP assessment to address complexities such as comorbidities, distress, and emerging frailty (for patients at high risk). GPs provided patient risk-stratification scoring or subgrouping alongside physiotherapy referrals. A GP training and support package lasted 2 h and included the intervention rationale, how it differs from usual care, familiarisation with the risk-stratification tool, its fit within the consultation, and discussion of questions or concerns. GPs also received a 1 h training update to share and discuss the first of their monthly feedback reports, showing individual GP intervention fidelity, with peer-to-peer comparisons.^20^

In the usual care group, patients with musculoskeletal pain consulting their GP received treatment as usual. This approach typically includes advice and education, medication, referral for investigations or tests, or referral to other services, such as physiotherapy, or secondary care specialists, such as orthopaedics and rheumatology, without the use of risk-stratification tools to support decision making. However, all patients completed the risk-stratification tool as part of the baseline and follow-up questionnaires, which were not seen by their GP, and these data were used for risk subgroup comparisons in the analysis.

### Outcomes

The primary outcome was time-averaged pain intensity over 6 months. This choice was informed by patient involvement during trial design, and a responsiveness analysis of pilot data showing sensitivity to change in this population. At the point of consultation and each monthly follow-up patients were asked: “How intense was your pain, on average, over the last 2 weeks?” (0–10 numerical rating scale). Secondary patient outcomes were collected at 6 months using self-completed postal questionnaires (appendix p 1); additional details are also in the published protocol.^13^ Information from participating GPs was collected on serious adverse events and adverse events. Process outcomes were collected to examine template use among musculoskeletal consultations and changes in GP clinical decision making, via a prospective 6-month anonymised medical record audit of all patients with an electronic study tag. The audit included: prescriptions (split by simple analgesics, non-steroidal anti-inflammatories, neuromodulators, muscle relaxants, and weak and strong opioids), referrals (split by physiotherapy or musculoskeletal interface clinic, specialist orthopaedics, pain clinic, and rheumatology), imaging for musculoskeletal disorders (split by x-ray or MRI, ultrasound, and bone density scan), sick certifications or fit notes, and repeat musculoskeletal GP visits over 6 months. The initial questionnaire also collected whether patients reported having received written advice or information from the GP about their condition. There were no changes to the trial outcomes after the trial commenced.

### Statistical analysis

We aimed to recruit 1200 participants (appendix p 19) from 24 general practices (equal clusters of 12 per group) to detect a standardised mean difference of 0·2 in time-averaged pain intensity over 6 months (primary outcome) with 90% power, an α of 5% (two-tailed), 25% dropout, and intra-class correlation for clustering of 0·01 at the GP practice level, allowing for a coefficient of variation in recruitment per practice of 0·65.^21^ This corresponded to a mean difference of 0·5 (for an anticipated SD of 2·5) on the pain-numeric rating scale.

All analyses were done by intention to treat (defined by general practice clusters), following the Consolidated Standards of Reporting Trials guidelines.^22^ No interim analyses were done.

The primary analysis compared mean differences in pain intensity scores between the trial groups over 6 months using a hierarchical linear mixed regression model evaluating repeated measures data at 1-month, 2-month, 3-month, 4-month, 5-month, and 6-month follow-up (level 1) within individuals (level 2) and considering clustering of individuals within general practices, the unit of randomisation (level 3). Secondary analyses used a linear mixed model for numerical outcomes and logistic mixed models for categorical outcomes at 6-month follow-up only. The analyses were adjusted for age, sex, and baseline pain intensity score (recorded at the point of consultation) at the individual-patient level, and general practice size. We also used treatment-by-time interaction terms to evaluate between-group differences in mean responses across each of the individual primary outcome time points of 1, 2, 3, 4, 5, and 6 months. Model fit was assessed across different covariance structures (unstructured, independence, exchangeable, and autoregressive) to ascertain the best-fit model.

Prespecified sensitivity analyses (per protocol, based on alternative definitions of good outcome, alternative assumptions about missing data and interval-censoring, and complete case analysis [those participants responding to all monthly follow-up]) were done to assess robustness of primary analyses. Prespecified subgroup analyses included additional interaction terms within the models for intervention group by risk subgroups (low [reference category], medium, and high risk), single musculoskeletal pain site (reference) versus multi-site pain, and pain site (back [reference], shoulder, knee, neck, and multi-site pain).

To examine differences in clinical decision making between trial groups, statistical testing for the use of the different treatment options between stratified versus usual care was done using negative binomial mixed models for count data, with practice (random factor) and practice size and participants' point-of-consultation pain score (fixed factors), except where logistic mixed modelling with the same fixed or random factors was used (for binary data, or due to lack of model convergence or small counts).

The primary economic evaluation was done from an NHS and personal social services perspective, with secondary analysis from health-care and societal perspectives. Resource use data, productivity loss, and changes in quality of life (EQ-5D-5L) required for the economic evaluation were collected from participants in 6-month follow-up questionnaires. Unit costs (in 2019 £) were obtained and used in accordance with standard sources and attached to resource use items.^23^, ^24^, ^25^ Utility data were generated using EQ-5D-5L participant responses from baseline and 6-month follow-up questionnaires, estimating quality-adjusted life-years (QALYs) for every participant, using the area under the curve approach, assuming linear interpolation between the measurements.^26^ We used a multi-level modelling statistical approach, taking into consideration clustering in cost and effect data and multiple imputation of missing data, to estimate the incremental cost per QALY gained for stratified care compared with usual care.^27^ Further details, including sensitivity and prespecified subgroup analyses, will be published separately.

Data collection for the nested qualitative study used semi-structured interviews with patients and focus groups and telephone interviews with clinicians in the stratified care arm (27 patients and 20 clinicians). Data were analysed thematically, and identified themes mapped onto the COM-B model^28^ and Normalisation Process Theory.^29^ Full qualitative methods and findings will be published separately.

Statistical analyses were done with SPSS version 24, Stata version 15, and R version 3.6.2. External trial steering and data monitoring committees oversaw the trial. The trial is registered with ISRCTN, ISRCTN15366334.

### Role of the funding source

The funder of the study had no role in study design, data collection, data analysis, data interpretation, or writing of the report. Patients with experience of musculoskeletal pain were involved in developing the funding application, the conduct of the trial (eg, reviewing patient facing documentation, deciding on the primary outcome, advising on interview topic guides, and encouraging the use of verbal consent before invitation letters being sent) and interpreting the trial results (eg, reviewing the qualitative data [reported separately] and discussing the interpretation of the findings).

## Results

**Figure 1:**
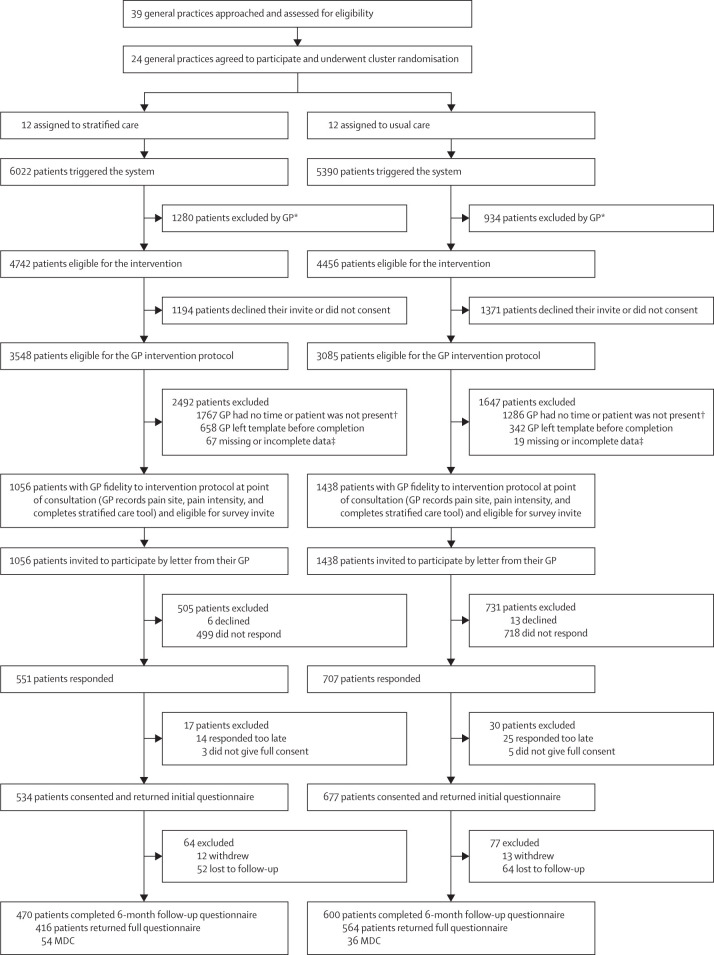
Trial profile GP=general practitioner. MDC=minimal data collection. NRS=numeric rating scale. *Not clinically relevant based on GP opinion (985 [16%] participants in the stratified care group, 643 [12%] in the usual care group), vulnerable patient (151 [3%] in the stratified care group, 151 [3%] in the usual care group), not trial-specific pain site consultation (50 [1%] in the stratified care group, 113 [2%] in the usual care group), and suspected serious pathology (94 [2%] in the stratified care group, 27 [1%] in the usual care group). †GP no time (1171 [19%] participants in the stratified care group, 695 [13%] in the usual care group) and patient not present (596 [10%] in the stratified care group, 591 [11%] in the usual care group). ‡Incomplete data (56 [1%] participants in the stratified care group, one [1%] participant in the usual care group) and IT processing error (11 [<1%] in the stratified care group, 18 [<1%] in the usual care group).

Between May 1, 2018, and April 30, 2019, 24 general practices (12 per study group; total adult practice size of 185 088 participants [96 397 in the stratified care group and 88 691 in the usual care group]; 104 GPs in total) participated from the West Midlands region of England. Trial recruitment templates were activated in 11 412 patients, of whom 9198 were eligible but 2565 declined to participate (figure 1). GP enrolment was completed in 2494 (38%) of 6633 consultations where trial protocols were possible (1056 [30%] stratified care and 1438 [47%] usual care). 1211 (49%) of 2494 participants provided consent and completed the initial questionnaire within 30 days (534 in the stratified care group and 677 in the usual care group). In the stratified care group, 2779 (87%) of a possible 3204 monthly pain NRS follow-up questionnaires were returned (482 participants gave three or more responses; 387 participants gave six responses). In the usual care group, 3654 (90%) of a possible 4062 monthly pain NRS follow-up questionnaires were returned (633 participants gave three or more responses; 519 participants gave six responses). For the primary analysis, if the last monthly brief questionnaire response was missing it was imputed using the corresponding pain response from the returned 6-month questionnaire (if completed within 20 days of the date of issue—this gave an overall number of available scores for the analysis of 2791 (87%) of 3204 for the stratified care group and 3668 (90%) of 4062 for the usual care group. The mean participant age was 60 years (range 18–95; SD 15·3) and 713 (59%) of 1211 participants were female (table 1). The mean pain intensity at the point of consultation was 6·73 (6·77 in the stratified care group and 6·70 in the usual care group). There were some differences in the characteristics of participants and non-participants as recorded by GPs at the point of consultation (appendix p 3). For example, the proportion of patients with different pain sites was significant (p=0·0072), mainly because of differences in back pain in the participants versus non-participants. However, the mean pain score and risk strata proportions for participants versus non-participants in the stratified care general practices were similar (appendix p 3). The population characteristics of general practices randomly assigned to each study group were similar (appendix p 4). Participant characteristics were similar across study groups, including the proportions in each risk stratum, overall musculoskeletal health status, physical function, and fear avoidance beliefs. Although there were some differences in baseline age, employment status, and pain site (table 1), these differences were modest and not considered to represent substantial selection bias.

**Table 1:** Baseline participant characteristics

			**Stratified care (n=534)**	**Usual care (n=677)**	**Indicative p value**
**Demographics**					
Age, years			57·8 (15·3)	61·8 (15·0)	0·0040
Sex					0·87
	Female		313 (59%)	400 (59%)	..
	Male		221 (41%)	277 (41%)	..
**Point of consultation with GP**					
Pain intensity overall (0-10 NRS)*			6·8 (1·9)	6·7 (2·0)	0·73
	Back		7·1 (1·8)	6·9 (1·9)	0·31
	Neck		6·9 (1·6)	6·7 (2·2)	0·56
	Shoulder		6·7 (1·8)	6·8 (1·9)	0·70
	Knee		6·2 (2·1)	6·4 (2·3)	0·58
	Multi-site		7·2 (1·9)	7·0 (1·5)	0·52
**Baseline questionnaire**					
Days between consultation and returning questionnaire			16·6 (27·3)	16·8 (28·0)	0·90
Race					0·40
	White		513/530 (96%)	659/675 (98%)	..
	Non-white		13/530 (2%)	11/675 (2%)	..
	Prefer not to say		4/530 (1%)	5/675 (1%)	..
Lives alone			91 (17%)	121 (18%)	0·94
Currently employed			275 (54%)	286 (44%)	0·0015
	Performance at work in the past 6 months of current workers (0–10 NRS)†		4·9 (3·1)	4·8 (3·1)	0·73
Performance at work in past 6 months of total study population (0–10 NRS)†			4·8 (3·2)	4·6 (3·2)	0·53
Time off work			99/303 (33%)	203/336 (31%)	0·80
	Number of days off		5 (2·5–15)	10 (4–20)	0·34
Need help with health literacy					0·58
	Never		434/526 (83%)	539/665 (81%)	..
	Rarely		44/526 (8%)	58/665 (9%)	..
	Sometimes		31/526 (6%)	42/665 (6%)	..
	Often		14/526 (3%)	18/665 (3%)	..
	Always		3/526 (1%)	8/665 (1%)	..
Pain area affected					0·0072
	Back		214 (40%)	243 (36%)	..
	Neck		61 (11%)	68 (10%)	..
	Shoulder		71 (13%)	59 (9%)	..
	Knee		157 (29%)	222 (33%)	..
	Multi-site pain		31 (6%)	85 (13%)	..
Pain intensity (0–10 NRS)* by pain area			6·3 (2·2)	6·4 (2·2)	0·78
	Back		6·6 (2·2)	6·5 (2·2)	0·88
	Neck		6·3 (2·0)	5·9 (2·2)	0·33
	Shoulder		6·6 (1·9)	6·8 (2·2)	0·91
	Knee		5·9 (2·3)	6·1 (2·4)	0·41
	Multi-site		6·5 (2·5)	6·8 (1·7)	0·60
Distress (0–10 NRS)‡			5·9 (2·6)	5·8 (2·6)	0·91
Confidence to manage (0–10 NRS)§			5·1 (2·5)	5·3 (2·6)	0·19
Pain duration					0·67
		<3 months	126/528 (24%)	180/674 (27%)	..
		3–6 months	106/528 (20%)	101/674 (15%)	..
		7–12 months	65/528 (12%)	86/674 (13%)	..
		1–2 years	64/528 (12%)	83/674 (12%)	..
		3–5 years	74/528 (14%)	88/674 (13%)	..
		6–10 years	34/528 (6%)	49/674 (7%)	..
	>10 years		59/528 (11%)	87/674 (13%)	..
Overall pain change (−5 to +5)¶			0·3 (2·1)	0·3 (2·0)	0·83
Number of previous episodes in last 3 years					0·052
		0	135/530 (25%)	125/675 (19%)	..
		1	69/530 (13%)	75/675 (11%)	..
		2–3	98/530 (18%)	133/675 (20%)	..
		4–9	70/530 (13%)	115/675 (17%)	..
	≥10		158/530 (30%)	227/675 (34%)	..
Previous surgery related to problem					0·16
	0		455/508 (90%)	564/660 (85%)	..
	1		38/508 (7%)	61/660 (9%)	..
	2		5/508 (1%)	24/660 (4%)	..
	≥3		10/508 (2%)	11/660 (2%)	..
Days of moderate activity in past week			2 (1–4)	2 (0–5)	0·72
Physical function‖					
	Back (RMDQ)		9·9 (5·8)	9·2 (5·6)	0·16
	Neck (NDI)		17·5 (8·7)	15·7 (8·7)	0·24
	Shoulder (SPADI-Disability subscale)		46·8 (24·1)	50·9 (26·1)	0·78
	Knee (KOOS-PS)		56·5 (15·2)	55·8 (17·9)	0·71
	Multi-site (SF12-PCS)		37·6 (8·8)	33·2 (10·3)	0·035
	Standardised function scale (overall mean 0, SD 1)		0·01 (0·97)	0·00 (1·02)	0·84
MSK-HQ**			28·7 (9·9)	29·5 (10·3)	0·60
STarT MSK tool (clinical version)††			7·1 (2·7)	7·0 (2·9)	0·97
STarT MSK (clinical version) risk subgroup					0·96
	Low risk (0–4 score)		98/503 (19%)	126/627 (20%)	..
	Medium risk (5–8 score)		238/503 (47%)	286/627 (46%)	..
	High risk (9–12 score)		167/503 (33%)	215/627 (34%)	..
Health-related quality of life (EQ-5D)‡‡			0·56 (0·23)	0·55 (0·24)	0·75
Fear avoidance beliefs (TSK-11)§§			25·4 (6·4)	24·8 (6·5)	0·26
Listed long term conditions					0·24
	0		184 (34%)	202 (30%)	..
	1		192 (36%)	247 (36%)	..
	2		103 (19%)	147 (22%)	..
	≥3		55 (10%)	81 (12%)	..
Perceived reassurance from GP consultation (RQ¶¶), total			58·7 (16·0)	59·8 (16·6)	0·69
	Data gathering		16·5 (4·3)	16·6 (4·6)	0·89
	Relationship building		16·7 (4·3)	16·9 (4·5)	0·75
	Generic		10·7 (5·0)	10·6 (5·2)	0·78
	Cognitive		14·9 (5·1)	15·5 (5·1)	0·38
Satisfaction with GP care in last 6 months					0·26
	Very satisfied		123/519 (24%)	190/672 (28%)	..
	Quite satisfied		173/519 (33%)	244/672 (36%)	..
	No opinion		149/519 (29%)	153/672 (23%)	..
	Not very satisfied		58/519 (11%)	73/672 (11%)	..
	Not at all satisfied		16/519 (3%)	12/672 (2%)	..
Preferential mode of follow-up					0·013
	Text		285 (53%)	310 (46%)	..
	Post		249 (47%)	367 (54%)	..

Data are mean (SD), n (%), or median (IQR). p values were derived through linear or generalised mixed models accounting for general practice clustering (random factor), and are for indicative purposes only. GP=general practitioner. KOOS-PS=Knee Injury and Osteoarthritis Outcome Score Physical Function Short-form. MSK-HQ=Musculoskeletal Health Questionnaire. NDI=Neck Disability Index. NRS=numeric rating scale. RMDQ=Roland-Morris Disability Questionnaire. RQ=Reassurance Questionnaire. SF12-PCS=Short Form 12v2 Physical Component Scale. SPADI=Shoulder Pain and Disability Index. TSK-11=Tampa Scale of Kinesiophobia.

* NRS-Pain: 0=no pain, 10=worst ever pain.

† Performance at work (0–10 NRS) where 0=problem not at all affected performance over last 6 months, 10=so bad I am unable to do my job.

‡ NRS-Distress: 0=no distress, 10=extreme distress.

§ NRS-Confidence to manage: 0=not at all confident, 10=extremely confident.

¶ Pain change scale 11-point NRS scale (−5 to +5) where −5=very much worse, 0=unchanged, +5=completely recovered (change from clinic appointment to time of self-report baseline completion).

‖ RMDQ (0–24 scale) where 0=no low back pain or disability, 24=maximum low back pain or disability; NDI (0–50 scale) where 0=no disability, 50=maximum disability; SPADI-Disability subscale (0–100): 0=no disability, 100=maximum disability; KOOS-PS (0–100): 0=extreme disability, 100=no disability. SF12-PCS (0–100): 0=worst physical health score, 100=best physical health score.

** MSK-HQ (0–56 scale) based on summation of 14 items on a 0–4 scale and where 0=worst musculoskeletal health-status and 56=best musculoskeletal health-status.

†† STarT MSK Tool score (0–12): 0=lowest risk, 12=highest risk.

‡‡ EuroQoL EQ-5D (−0·59 to 1·00): −0·59=worst health status, 1·00 best health status.

§§ TSK-11 (11–44): 11=minimum fear avoidance, 44=maximum fear avoidance.

¶¶ RQ (12–84 scale): 12=no reassurance, 84=high reassurance (subscales all recorded on 3–21 scale: 3=no reassurance, 21=high reassurance)

**Figure 2:**
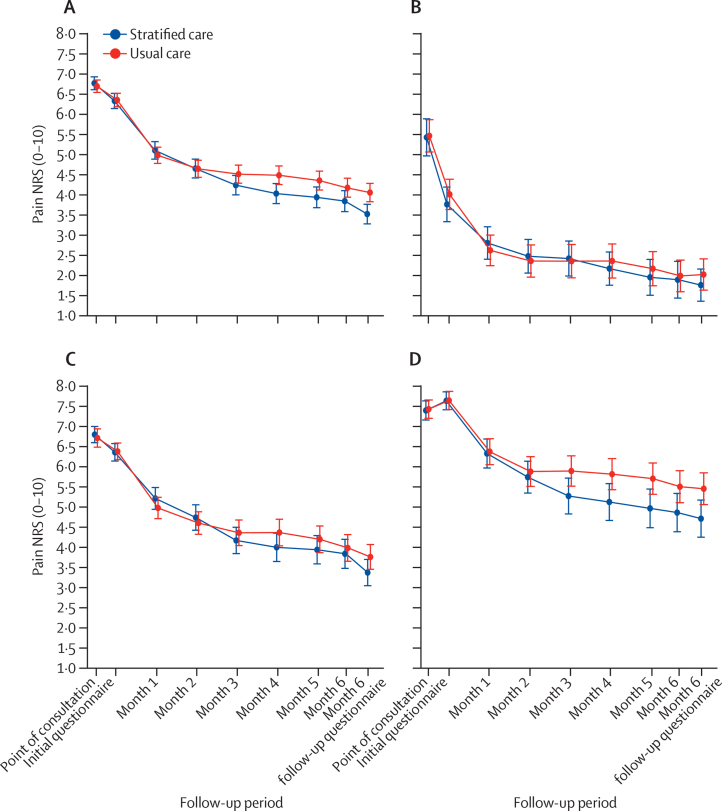
Mean monthly pain intensity scores (A) All participants. (B) Low-risk participants. (C) Medium-risk participants. (D) High-risk participants. NRS=numeric rating scale. Mean average and mean differences in pain scores for all participants can be found in the appendix (p 5) and for each patient risk subgroup (p 11). Error bars indicate 95% CIs.

The primary outcome of time-averaged pain intensity over 6 months was available for 1178 (97%) of 1211 participants (515 [96%] of 534 participants in the stratified care group and 663 [98%] of 677 participants in the usual care group) and the full 6-month questionnaire was completed by 1070 (88%) of 1211 participants (470 [88%] of 534 participants in the stratified care group and 600 [89%] of 677 participants in the usual care group). In the primary analysis, we found no statistically significant differences in time-averaged pain intensity between the study groups, with mean 4·4 (SD 2·3) for stratified care and 4·6 (2·5) for usual care (figure 2). The adjusted mean difference was −0·16 (95% CI −0·65 to 0·34; p=0·535), translating to a standardised mean difference (effect size) of −0·08 (−0·33 to 0·17; table 2). Mean differences in pain intensity were consistently greater in the latter 3 months than the first 3 months, although the average mean difference over months 4 to 6 was also not statistically significant (−0·33, −0·84 to 0·19; p=0·211). Most sensitivity analyses showed no between-group differences, despite showing consistent slightly favourable results for risk-based stratified care (appendix p 13). Analysis of minimal clinically important change (more than one point change in pain intensity) gave an overall odds ratio (OR) of 1·66 (95% CI 0·98 to 2·82; p=0·061). We found a significant difference (OR 2·22, 95% CI 1·26 to 3·89; p=0·0063) for the 4 to 6 months comparison (appendix p 5). We did a post-hoc analysis of our primary outcome by sex (appendix p 14), which showed a significant positive effect on pain intensity from risk-based stratified care in male participants (mean difference −0·67, 95% CI −1·27 to −0·07) but not in female participants (0·14, −0·42 to 0·69; p_interaction_=0·019). The trial was not powered for this post-hoc analysis and so these results should be treated with caution. No serious adverse events or adverse events were reported.

**Table 2:** Mean monthly pain intensity scores per group (primary outcome)

	**Patients analysed in the stratified care group**	**Patients analysed in the usual care group**	**Stratified care pain intensity**	**Usual care pain intensity**	Mean difference*(95% CI)	**Standardised mean difference**†**(95% CI)**	**p value**
Point of consultation	534	675	6·8 (1·9)	6·7 (2·0)	..	..	..
Baseline	533	675	6·3 (2·2)	6·4 (2·2)	..	..	..
Month 1	491	632	5·1 (2·5)	5·0 (2·6)	0·17 (−0·34 to 0·68)	0·09 (−0·17 to 0·35)	0·51
Month 2	470	623	4·7 (2·6)	4·6 (2·7)	0·09 (−0·43 to 0·60)	0·05 (−0·22 to 0·31)	0·74
Month 3	478	615	4·2 (2·7)	4·5 (2·8)	−0·21 (−0·73 to 0·30)	−0·11 (−0·37 to 0·15)	0·42
Month 4	455	604	4·0 (2·7)	4·5 (2·9)	−0·40 (−0·92 to 0·13)	−0·21 (−0·47 to 0·07)	0·14
Month 5	451	601	3·9 (2·8)	4·4 (2·9)	−0·35 (−0·88 to 0·18)	−0·18 (−0·45 to 0·09)	0·20
Month 6‡	446	593	3·8 (2·8)	4·2 (2·9)	−0·24 (−0·78 to 0·30)	−0·12 (−0·40 to 0·15)	0·39
Mean (1–6 months)§	515	663	4·4 (2·3)	4·6 (2·5)	−0·16 (−0·65 to 0·34)¶	−0·08 (−0·33 to 0·17)	0·54
Mean (4–6 months)‖	486	642	4·0 (2·6)	4·3 (2·7)	−0·33 (−0·84 to 0·19)¶	−0·17 (−0·43 to 0·10)	0·21

Data are n or mean (SD), unless otherwise indicated.

* Between-group difference in mean scores (stratified care – usual care) by linear mixed model with practice and participants (random factors) and practice size and participants' age, gender, and (point-of-consultation) pain score (fixed factors).

† Relative to numeric rating scale pain point-of-consultation SD (1·95).

‡ If the last monthly brief questionnaire response was missing it was imputed using the corresponding pain response from the returned 6-month questionnaire (if completed within 20 days of the date of issue).

§ Primary endpoint (mean of available data for 1–6 months follow up). Average summary mean (SD) relates to the mean of available 1–6 month follow-up data. Contrast group × time: χ^2^ test 19·8; five degrees of freedom; p=0·0014.

¶ Kenward-Roger/Satterthwaite adjustment to CI: mean 1–6 months (−0·68 to 0·37); mean 4–6 months (−0·87 to 0·22).

‖ Post-hoc analysis (not prespecified).

There were no statistically significant differences in secondary clinical outcomes at 6 months, except for a significantly larger improvement in shoulder pain and function and higher satisfaction with care in the stratified care arm (table 3). We found no differences in mean monthly measured levels of psychological distress or pain self-efficacy (appendix p 12). Prespecified exploratory subgroup analyses showed larger between-group mean differences (although not statistically significant) in patients at high risk versus low risk and medium risk (figure 2; appendix p 11), and between those with shoulder and knee pain compared with those with neck and back pain (appendix pp 6–7).

**Table 3:** Secondary outcome measures at 6 months

		**Stratified care (n=534)**	**Usual care (n=677)**	Mean difference or OR (95% CI)*	**p value**
**Mean differences**					
Pain intensity reported in questionnaire (0–10 NRS)†		3·5 (2·7)	4·1 (2·8)	−0·45 (−0·97 to 0·07)	0·088
Overall global change (−5 to +5)‡		1·7 (2·4)	1·2 (2·6)	0·29 (−0·10 to 0·68)	0·14
Days of moderate activity in last week		3·2 (2·1)	3·2 (2·3)	−0·06 (−0·35 to 0·22)	0·66
Physical function§					
	Back (RMDQ)	6·4 (5·7)	6·1 (5·5)	−0·30 (−1·30 to 0·70)	0·56
	Neck (NDI)	11·5 (8·9)	12·3 (9·1)	−1·01 (−4·81 to 2·80)	0·60
	Shoulder (SPADI-Disability subscale)	25·5 (27·3)	39·5 (31·4)	−11·10 (−19·80 to −2·30)	0·013
	Knee (KOOS-PS)	68·1 (14·7)	65·6 (20·0)	0·35 (−4·94 to 5·64)	0·90
	Multi-site (SF12-PCS)	40·4 (9·9)	35·8 (11·5)	0·31 (−4·40 to 5·01)	0·90
	Standardised function score	−0·06 (0·94)	0·05 (1·04)	−0·07 (−0·22 to 0·08)	0·34
MSK-HQ¶		39·2 (11·0)	37·4 (12·1)	1·57 (−0·30 to 3·45)	0·10
STarT MSK tool (clinical version)‖		4·8 (2·8)	5·1 (3·1)	−0·27 (−0·73 to 0·20)	0·27
Health-related Quality of Life (EQ-5D)**		0·67 (0·23)	0·65 (0·24)	0·022 (−0·003 to 0·048)	0·082
Fear avoidance beliefs (TSK-11)††		22·7 (7.0)	23·3 (7·4)	−0·64 (−1·70 to 0·42)	0·24
Performance at work over last 6 months (0–10 NRS)‡‡		3·4 (2·8)	3·5 (3·0)	−0·18 (−0·62 to 0·27)	0·43
**ORs**					
STarT MSK (clinical version) risk subgroup		..	..	0·76 (0·51 to 1·13)	0·17
	Low risk (0–4 score)	211/398 (53%)	263/541 (49%)	..	..
	Medium risk (5–8 score)	136/398 (34%)	180/541 (33%)	..	..
	High risk (9–12 score)	51/398 (13%)	98/541 (18%)	..	..
Currently employed		177/410 (44%)	219/541 (40%)	0·83 (0·43 to 1·59)	0·57
Time off work in last 6 months		47/200 (22%)	60/245 (24%)	0·97 (0·53 to 1·78)	0·92
Satisfaction with care		..	..	0·74 (0·57 to 0·98)	0·033
	Very satisfied	125/421 (30%)	138/551 (25%)	..	..
	Quite satisfied	141/421 (34%)	205/551 (37%)	..	..
	No opinion	97/421 (23%)	134/551 (24%)	..	..
	Not very satisfied	39/421 (9%)	64/551 (11%)	..	..
	Not at all satisfied	11/421 (3%)	18/551 (3%)	..	..

Data are mean (SD) or n (%), unless otherwise indicated. KOOS-PS=Knee Injury and Osteoarthritis Outcome Score Physical Function Short-form. MSK-HQ=Musculoskeletal Health Questionnaire. NDI=Neck Disability Index. NRS=numeric rating scale. OR=odds ratio. RMDQ=Roland-Morris Disability Questionnaire. SF12-PCS=Short Form 12v2 Physical Component Scale. SPADI=Shoulder Pain and Disability Index. TSK-11=Tampa Scale of Kinesiophobia.

* Between-group difference in mean scores (stratified care – usual care) and OR (stratified care *vs* usual care) respectively by linear and generalised mixed models with practice (random factor) and practice size and participants' age, gender, and (point-of-consultation) pain score and corresponding baseline measure response if available (fixed factors).

† NRS-Pain: 0=no pain, 10=worst ever pain.

‡ Pain change scale 11-point NRS scale (−5 to +5) where −5=very much worse, 0=unchanged, +5=completely recovered (change from clinic appointment to time of self-report baseline completion).

§ RMDQ (0–24 scale) where 0=no low back pain or disability, 24=maximum low back or pain disability; NDI (0–50 scale) where 0=no disability, 50=maximum disability; SPADI (0–100): 0=no disability, 100=maximum disability); KOOS-PS (0–100): 0=extreme disability, 100=no disability; SF12-PCS (0–100): 0=worst physical health score, 100=best physical health score.

¶ MSK-HQ (0–56 scale) based on summation of 14 items on a 0–4 scale and where 0=worst musculoskeletal health status and 56=best musculoskeletal health status.

‖ STarT MSK Tool score (0–12): 0=lowest risk, 12=highest risk.

** EuroQoL EQ-5D (−0·59 to 1·00): −0·59=worst health status, 1·00 best health status.

†† TSK-11 (11–44): 11=minimum fear avoidance, 44=maximum fear avoidance.

‡‡ Performance at work (0–10 NRS) where 0=problem not at all affected performance over last 6 months, 10=so bad I am unable to do my job.

GPs in stratified care practices completed the risk-stratification tool in 1056 (30%) of 3548 possible consultations and reported selecting an appropriate risk-matched treatment option in 815 (77%) patients (176 [78%] of 277 low risk, 457 [78%] of 585 medium risk, and 182 [75%] of 244 high risk). Full intervention fidelity details are in the appendix (p 10). An anonymised medical record audit (n=2494) showed that stratified care led to some significant intended changes in clinical decision making compared with usual care at the point of consultation (appendix pp 15–18), increased provision of written information, physiotherapy referral, and simple over-the-counter analgesic medication. However, an unintended effect was also observed, as the prescribing of short-term courses of strong opioids increased, although this was limited to the index GP consultation and long-term opioid prescribing remained unchanged at subsequent consultations.

Economic evaluation showed that costs of care were very similar in both trial groups; the adjusted incremental cost of stratified care compared with usual care over 6 months was £6·85 (95% CI −107·82 to 121·54), with incremental QALYs of 0·0041 (95% CI −0·0013 to 0·0094), representing a net QALY gain. Stratified care was associated with an incremental cost-effectiveness ratio of £1671 per additional QALY gained. At a willingness-to-pay threshold of £20 000 per QALY, the incremental net monetary benefit was £132 and the probability of stratified care being cost-effective was approximately 73%. Furthermore, the stratified care intervention produced a small incremental gain in quality of life compared with usual care for a minimal increase in cost. Although the findings suggest that risk-based stratified care is potentially a cost-effective use of health-care resources when applying conventional rules of cost-effectiveness, the very small differences suggest that caution should be taken in the interpretation of this result.

Within the embedded qualitative study findings, many GPs reported that stratified care had a positive role in informing their clinical decision making, including giving greater attention to psychosocial issues (particularly for shoulder and knee pain, for which they previously took a more biomechanical approach), taking a more functional approach, and facilitating negotiations with patients about options such as imaging. Patients reported that the STarT MSK Tool items had added value, and in particular, questions about mood were seen to facilitate a more holistic approach. Physiotherapists found the additional STarT MSK Tool information useful, but there were no other changes reported in GP or physiotherapist interprofessional communication.

## Discussion

To our knowledge, this is the first large clinical trial of risk-based stratified care for a broader group of patients with musculoskeletal pain in primary care. No significant benefits were shown for the primary outcome of time-averaged pain intensity over 6 months, nor for most secondary outcomes, with the exception of patient satisfaction with care and shoulder function. However, risk-based stratified care at the point of consultation did significantly change some aspects of GP decision making as intended, leading to greater provision of written information and prescribing of simple over-the-counter analgesics. At the same time, we also observed a negative and unintended increase in short-term prescribing of strong opioids and a high rate of referral to physiotherapy for low-risk patients. Overall, the costs of care were similar, as the four times overall increase in physiotherapy referral over 6 months was balanced by health-care savings in imaging and secondary care referrals. The qualitative findings highlighted positive GP feedback, suggesting the approach improved their consideration of psychosocial factors and decision making, and helped with negotiations around patient expectations, such as unwarranted imaging.

The findings of this trial contrast with our previous successful stratified care trial in patients with low back pain in the UK.^10^, ^30^ Potential reasons for this include the low GP fidelity to using the risk tool and a lack of effectiveness of the risk-matched treatments. We did not see evidence that low GP uptake was due to difficulties in accessing or using the interface, perhaps because a strength of the intervention template was that it was embedded into the existing routinely used record system and fired automatically when a relevant diagnostic code was entered. We believe that low GP uptake was more likely to be a result of the timing of the template trigger, which often occurred after the patient had left the room, and because of the current time-pressured context of UK primary care and GPs feeling the stratification tool added time to the consultation. This was evidenced by GPs stating they did not have time or that the patient was not present in half of potentially suitable musculoskeletal pain consultations. This study was conducted before the COVID-19 pandemic and so typically patients were not present because they had left the room before the GP began to enter the consultation details into the medical record. We would also note that after our pilot trial,^15^ in which GP fidelity to completing the tool occurred in 32% of eligible consultations, we revised our expectation for GP fidelity from 50% to 25% of coded musculoskeletal pain consultations, mainly because GPs reported the intervention was only appropriate where musculoskeletal pain was the primary problem for the visit, and they felt the tool often fired when the pain was presenting as a comorbid condition. The unintended increase in prescribing of short-term strong opioids among intervention GPs was unexpected and was the opposite of what we observed in our pilot trial, in which opioid prescribing reduced. These differences are likely to relate to a change to our risk-matched treatment options, as in the pilot trial opioids were only recommended for high-risk patients, whereas in the main trial weak opioids were a recommended treatment option for medium-risk patients as well. In the prespecified but not powered subgroup analysis, stratified care was less successful for back, neck, and multi-site pain than for shoulder and knee pain. A potential reason for this difference that emerged from the qualitative study is that intervention GPs reported the prognostic tool encouraged them to be more holistic in consultations for shoulder and knee pain, whereas they felt they already used a holistic approach for spinal and multi-site pain. It is noteworthy that the small differences observed between study groups started at around the 3-month follow-up, which is likely to be when patients began receiving NHS physiotherapy, as there was a 6–8 week waiting list at the time of the trial. In this trial, we did not upskill physiotherapists or optimise clinical pathways to deliver risk-matched treatments such as three-day physiotherapy training for medium risk and six-day training programmes (and ongoing regular mentoring) on psychologically informed physiotherapy for patients at high risk of poor outcome. In the STarT Back trial, the intervention mean change in Roland Morris Disability Questionnaire at 4 months was 4·7 versus 3·0 in the control group. In the present trial, back pain function improved by much less at 6 months (intervention 3·5 *vs* control 3·1). To our knowledge, no other stratified care trials for low back pain, to date, have provided as intense a training and mentoring programme as the STarT Back Trial.^30^ Other successful primary care risk prediction tools, such as QRISK3 for estimating cardiovascular risk, have the benefit of highly effective pharmaceutical treatment for those identified as being at increased risk. Therefore, the key challenge for future trials of risk-based stratified care among patients with musculoskeletal pain is, first, to find more feasible methods to stratify patients in short consultations and, second, to provide more effective treatments for those at increased risk. Without overcoming these challenges, trials risk becoming a test of stratified care implementation rather than effectiveness. The findings of this trial highlight that although early risk identification in a small proportion of patients positively changes some aspects of primary care decision making, without more effective matched treatments for those at increased risk, benefits at the level of patient outcomes are unlikely.

Strengths of this trial include the large sample size, high follow-up rates for the primary outcome, robust process assessment, and embedded health economic and qualitative studies. A further strength was that the medical record review of clinical decision making included all adults for whom there was a musculoskeletal consultation, not only those who consented to data collection via questionnaires. Limitations of our trial include no data capture on additional care from the referral destination services, and that the trial was not powered to detect differences at the level of each risk stratum, and therefore the conclusions only apply to the overall stratified primary care approach versus usual care. There are some limitations affecting the generalisability of the trial. The setting of the trial was in the West Midlands region of England, which is the second most ethnically diverse region in the UK and is known to have relatively high economic hardship, with historically higher levels of unemployment than other regions of the UK. Although the index of multiple deprivation scores among intervention and control general practices were similar, these economic factors relating to the location of the trial might have skewed outcomes, such as return to work rates and compliance with care regimens in ways not found in other UK regions. The multiplicity of analyses is also a limitation, and we suggest caution particularly around the interpretation of the post-hoc analysis, such as the finding of significant differences between the effects of stratified care in male (significant improvement) and female (non-significant improvement) participants. At present the reasons for this finding are unknown and further research is needed.

As this was a cluster-randomised trial, testing of baseline characteristics between groups was completed to check for potential imbalances that might have occurred at the cluster level, and which could have led to significant imbalances at the individual level that the analysis was based on.^31^ In the future, we recommend that an automated medical record risk identification method is used in primary care to overcome challenges around clinicians using risk tools during short and busy consultations; that high quality, evidence-based treatments are provided for patients at high risk, including clinicians having additional training and skills development to better manage complex musculoskeletal patients; and that simpler stratified care models are used, which might be easier to implement (ie, 15 treatment options in this trial was too complex).

In conclusion, our results show that risk-based stratified care in general practice for patients with common musculoskeletal pain presentations does not lead to significant improvements in patient outcomes, despite some benefits to GP decision making and positive GP and patient experiences of care. Although the economic evaluation suggests risk-based stratified care could offer a cost-effective use of health-care resources, the minimal difference in costs and outcomes suggests that caution should be taken in the interpretation of this result.

## Data sharing

Metadata, including the study protocol, statistical analysis plan, data dictionaries, and key study documents (patient information leaflet, blank or coded case report forms, and consent form), will be deposited on a publicly accessible repository. De-identified individual participant data that underlie the results from this trial will be securely stored on servers approved by a government-backed cyber security scheme and made available to bona-fide researchers upon reasonable request via our controlled access procedures. Unless there are exceptional circumstances, data will be available upon publication of the main trial findings and with no end date. Data requests and enquiries should be directed to primarycare.datasharing@keele.ac.uk. We encourage collaboration with those who collected the data, to recognise and credit their contributions. The data generated from this trial will remain the responsibility of the study sponsor. Release of data will be subject to a data use agreement between the sponsor and the third party requesting the data. De-identified individual patient data will be encrypted upon transfer.

## Declaration of interests

JCH, NEF, ML, KMD, JP, DvdW, CM, and EMH report grants from the National Institute for Health and Care Research. All other authors declare no competing interests. This paper presents independent research funded by the National Institute for Health Research (NIHR) under its Programme Grants for Applied Research scheme (grant number RP-PG-1211-20010); and Versus Arthritis through a Centre of Excellence grant (grant reference 20202). Nadine Foster is a NIHR Senior Investigator and was supported through an NIHR Research Professorship (NIHRRP-011-015). The views expressed in this publication are those of the authors and not necessarily those of the UK National Health Service, the NIHR, or the Department of Health and Social Care. Funders of the study had no role in the design of the study, data collection, analysis and interpretation of data, or in writing the manuscript.
