# Supplementary material for: Risk-based stratified primary care for common musculoskeletal pain presentations (STarT MSK): a cluster-randomised, controlled trial
Source: Lancet Rheumatol. 2022 Jul 15;4(9):e591–602. doi: 10.1016/S2665-9913(22)00159-X (PMC9649927; doi:10.1016/S2665-9913(22)00159-X)
Supplement: Supplementary appendix [file mmc1.pdf]

THE LANCET
Rheumatology

Supplementary appendix

This appendix formed part of the original submission and has been peer reviewed. We post it as supplied by the authors.

Supplement to: Hill JC, Garvin S, Bromley K,et al. Risk-based stratified primary care for common musculoskeletal pain presentations (STarT MSK): a cluster-randomised, controlled trial. *Lancet Rheumatol* 2022; published online July 15. https://doi.org/10.1016/S2665-9913(22)00159-X.

Appendices

Appendix 1: Primary and secondary self-reported outcomes and collection timepoints

Conceptual domain	Operational definition	Empirical measure used	Number of items	Time-point of data collection
**Patient descriptors**				
Age	Age at index consultation	Date of birth	1	GP EMR
Sex	Sex	Male / Female	1	GP EMR
Index pain location	Site of index pain complaint	Choice of anatomical region	1	GP EMR
Pain intensity	Usual pain intensity	NRS 0-10	1	GPEMR, I, 6FU, MF, MDC
Socioeconomic status (IMD)	The individual's (i) current or (ii) most recent job title	Job title - categorised as manual/non-manual	2	GP EMR
GP Practice	GP Practice consulted for MSK pain	Taken from medical record	1	GP EMR
Episode duration	Time since last whole month pain free	Episode duration	1	I
Health Literacy Screen	Health literacy	Single question - Likert scale	1	I
Comorbidities	Self-reported diagnosed comorbidities from a provided list	Yes	1	I
Widespread pain	Presence of widespread pain	Yes / no	1	I
Support needed	Support to complete questionnaire	Yes / no	1	I
Living arrangements	Lives alone	Yes / no	1	I
Previous episodes	Number of previous pain episodes	Number	1	I
Perceived reassurance from GP consultation	Effective Consultation and Reassurance Questionnaire (ECRQ)	12 items with 7-point Likert scale	12	I
Receipt of written education material from GP	Single item to ask if patient received written information at their GP visit	Yes / no / don't remember	1	I
Pain self-efficacy	Single item - confidence to manage pain	NRS 0-10	1	I, MF
Psychological distress	Single item regarding level of distress	NRS 0-10	1	I, MF
Employment status and absence from work	Employment status at time of questionnaire	Yes/No and details	1	I, 6FU
Risk status – development version of STarT MSK Tool	Risk of persistent disabling pain	Yes / No	9	I, 6FU
Musculoskeletal health	Impact from MSK symptoms	MSK-HQ	14	I, 6FU
Overall rating of change	Change since index pain consultation	Single question -5 to +5 scale	1	I, 6FU
Physical activity level	Days past week of moderate activity	1-7 days	1	I, 6FU
Fear avoidance beliefs	Fear of movement	TSK-11	11	I, 6FU
Satisfaction	Satisfaction with care	Single question - Likert scale	1	I, 6FU
Physical function				
Back pain patients	Site specific physical function	RMDQ – original version	24	I, 6FU
Neck pain patients		NDI	10	I, 6FU
Shoulder pain patients		SPADI	13	I, 6FU
Knee pain patients		KOOS-PS	7	I, 6FU
Multi-site pain		SF-12 PCS	12	I, 6FU
Health-related quality of life	Utility-based quality of life	EuroQol-5D	5	I, 6FU MDC
Healthcare costs				
Performance at work	How productivity at work is affected	0-10 NRS	1	I, 6FU
Work absence	Number of days absent from work	Yes/No and details	1	I, 6FU
Health care resource use	Use of primary care, other NHS services, and private healthcare	Yes/No and if Yes details of resources used	3	6FU

GP EMR – GP EMR audit; I – initial participant questionnaire; 6FU – 6-month participant follow-up questionnaire; NRS – numerical rating scale. MF – monthly participant follow-up questionnaire. MDC – minimal data collection. Supporting references are in the pilot trial publication.14

Appendix 2: Statistical analysis plan

See page 19

Appendix 3: Characteristics of participants versus non-participants.

	Participants (1211)	Non-participants (1283)	All invited patients (2494)
Trial Arm, n (%)			P=0.085
Stratified Care	534 (44.1)	522 (40.7)	1056 (42.3)
Usual Care	677 (55.9)	761 (59.3)	1438 (57.7)
Pain site, n (%)			P=0.004
Back	457 (37.7)	584 (45.5)	1041 (41.7)
Neck	129 (10.7)	151 (11.8)	280 (11.2)
Shoulder	130 (10.7)	117 (9.1)	247 (9.9)
Knee	379 (31.3)	327 (25.5)	706 (28.3)
Multi-site	116 (9.6)	104 (8.1)	220 (8.8)
Pain score, mean (SD)	6.7 (2.0)	6.8 (1.9)	P=0.12
**Stratified Care arm only**	**Participants (534)**	**Non-participants (522)**	**All invited patients in the stratified care arm (1056)**
Treatment as per protocol[#], n (%)			P=0.25
No	114 (21.4)	127 (24.3)	241 (22.8)
Yes	420 (78.6)	395 (75.7)	815 (77.2)
Risk subgroup, n (%)			P=0.050 (overall test) P=0.13 (trend test)
Low risk	99 (18.5)	128 (24.5)	227 (21.5)
Medium risk	311 (58.2)	274 (52.5)	585 (55.4)
High risk	124 (23.2)	120 (23.0)	244 (23.1)
STarT MSK Tool score, mean (SD)	6.7 (2.4)	6.4 (2.5)	P=0.085 6.5 (2.4)

P-values by chi square test (test for trend as indicated) for categorical variables and t-test for numerical variables.
Treatment as per protocol based on data GPs entered onto the trial-specific electronic template.

Appendix 4: General Practice characteristics per trial arm

Key characteristics	Stratified Care (n=12)	Usual Care (n=12)
Registered population size,		
- mean (SD)	8033 (3214)	7391 (3574)
- median	7361.5	6981.5
- IQR	(6277-9954.5)	(5616.5, 8628)
- minimum, maximum	1994 - 13248	2031 - 13894
Index of Multiple Deprivation (IMD), median (IQR)		
Income deprivation	19390.5 (14171, 25490.5)	17483.5 (10423.5, 25405.5)
Employment deprivation	14676 (11254.5, 24009.5)	14010 (9897, 24563)
Education, Skills & Training deprivation	20409 (10228, 28632.5)	18106 (8492.5, 25586.5)
Health deprivation & Disability	15656 (9502, 24854)	17587.5 (12628, 22859.5)
Crime	15095 (5253.5, 24809.5)	19234.5 (12500.5, 26251.5)
Barriers to Housing & Services	23271.5 (17355.5, 26325.5)	26846.5 (19260, 30273)
Living Environment deprivation	9448 (2717, 25406.5)	15491 (7144.5, 21202.5)
Index of Multiple Deprivation [IMD]	17995 (11240, 23126.5)	15626 (11146, 24248.5)

* English Indices of Area Deprivation: The 32,844 Lower-layer-Super-Output Areas in England are ranked according to their deprivation score. For each of the neighbourhood-level Indices, the most deprived Lower-layer Super Output Area in England is given a rank of 1, and the least deprived a rank of 32,844 (IMD is a weighted aggregate of the seven sub-indices).

Appendix 5: Number (%) achieving the MCIC for Pain Intensity NRS (at least 1-point change)

**Month**	**Stratified Care n (%)**	**Usual Care n (%)**	**OR (95% CI)#**	**P-value**	**% difference (95% CI)##**	**NNT (95% CI)**
1	319/491 (65.0%)	404/632 (63.9%)	0.89 (0.48, 1.66)	0.72	-2.7 (-18.0, 10.7)	36.6^{UC} (5.6^{UC}, 9.3^{SC})
2	332/470 (70.6%)	418/623 (67.1%)	1.11 (0.59, 2.10)	0.74	2.3 (-12.5, 14.0)	44.2^{SC} (8.0^{UC}, 7.2^{SC})
3	361/478 (75.5%)	414/615 (67.3%)	1.92 (1.01, 3.67)	**0.047**	12.5 (0.2, 21.0)	8.0^{SC} (457^{SC}, 4.8^{SC})
4	346/455 (76.0%)	397/604 (65.7%)	2.47 (1.29, 4.74)	**0.007**	16.9 (5.5, 24.4)	5.9^{SC} (18.2^{SC}, 4.1^{SC})
5	349/451 (77.4%)	408/601 (67.9%)	2.17 (1.12, 4.20)	**0.021**	14.2 (2.4, 22.0)	7.0^{SC} (41.3^{SC}, 4.5^{SC})
$6^{\$}$	347/446 (77.8%)	412/593 (69.5%)	2.03 (1.04, 3.94)	**0.037**	12.7 (0.8, 20.5)	7.9^{SC} (121^{SC}, 4.9^{SC})
Average 1-6 months^	371/515 (72.0%)	434/663 (65.5%)	1.66 (0.98, 2.82)	**0.061**	10.4 (-0.5, 18.8)$^{\$}$	9.6^{SC} (218^{UC}, 5.3^{SC})$^{\$}$
Average 4-6 months~^^	367/486 (75.5%)	423/642 (65.9%)	2.22 (1.26, 3.89)	**0.006**	15.2 (5.0, 22.4)$^{\$}$	6.6^{SC} (20.0^{SC}, 4.5^{SC})$^{\$}$

MCIC = Minimum Clinically Important Change.

P-values in **Bold** show significant differences in favour of stratified care over usual care.

Between-group comparison (odds ratio of Stratified Care to Usual Care) by generalised mixed model (logit link function) of long-dataset with practice and participants (random factors) and practice size and participants' age, gender and (point-of-consultation) pain score (fixed factors).

Absolute percentage difference derived from the odds ratio using the observed proportion in the control arm as the reference.

NNT = Number Needed to Treat (derived as the reciprocal of the % difference) where SC superscript denotes the NNT in respect of favourable Stratified Care arm and UC superscript denotes the NNT in respect of favourable Usual Care arm.

$ If the last monthly SMS/brief questionnaire response was missing it was imputed using the corresponding pain response from the returned 6-month questionnaire (if completed within 20 days of the date of issue).

Note: Contrast Arm x time: chi square test 20.3; 5 degrees of freedom; p=0.0011.

~ Post-hoc analysis (not pre-specified).

^ Sensitivity analysis using different cut-points (1-6 months)

(i) MCIC 2- point change OR=1.34 (0.75, 2.42) [P=0.322]; (ii) MCIC 3- point change OR=1.28 (0.71, 2.32) [P=0.410].

^^ Sensitivity analysis using different cut-points (4-6 months)

(i) MCIC 2- point change OR=1.87 (1.01, 3.43) [P=0.045]; (ii) MCIC 3- point change OR=1.88 (1.02, 3.48) [P=0.044].

Appendix 6a: Subgroup analyses by pain site (exploratory)

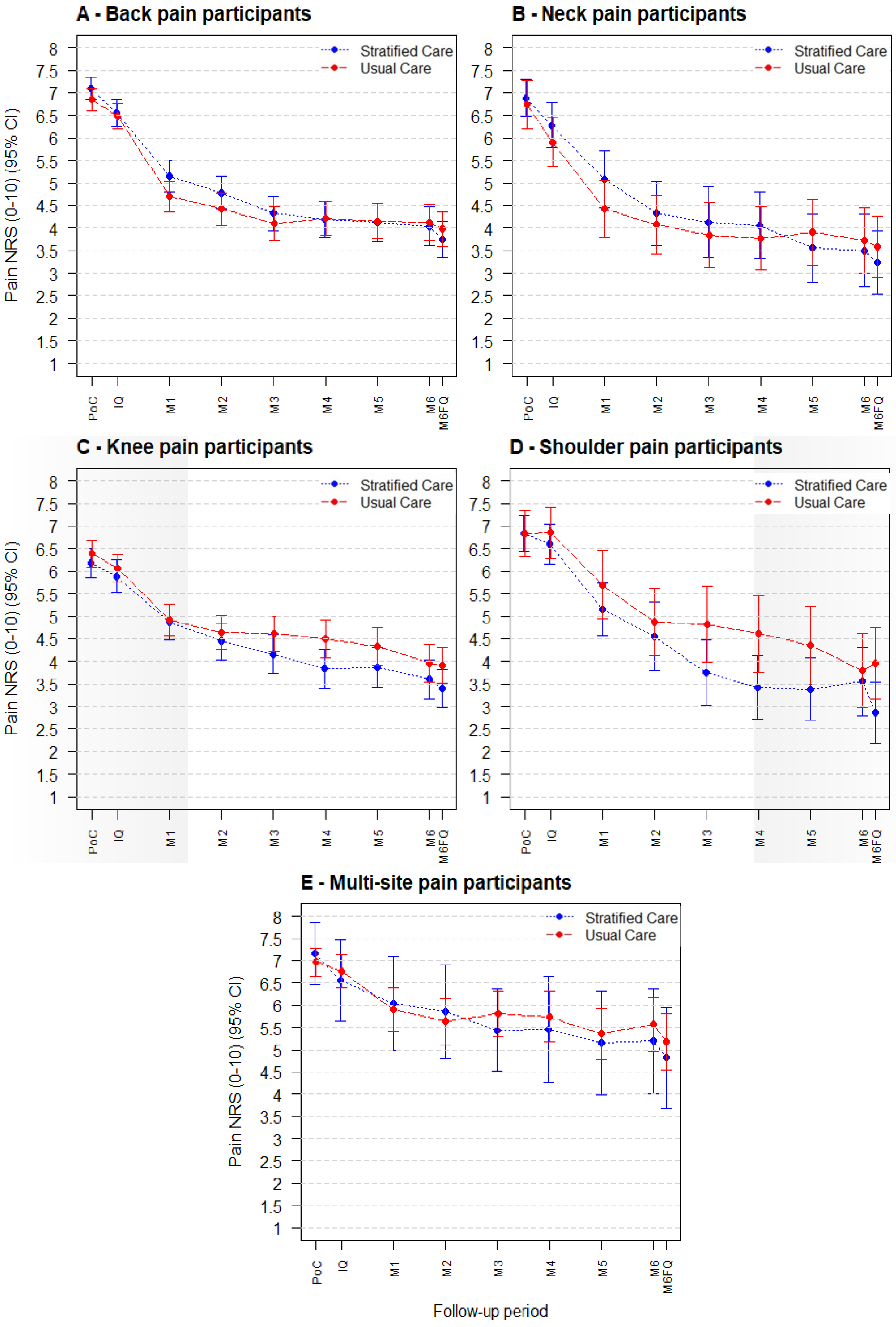

Appendix 6b:

Overall and Monthly Pain Intensity (NRS) scores per subgroup of musculoskeletal pain site

	Back		Neck		Shoulder		Knee		Multi-Site	
**Month**	Stratified Care	Usual Care	Stratified Care	Usual Care	Stratified Care	Usual Care	Stratified Care	Usual Care	Stratified Care	Usual Care
1	195, 5.2 (2.5)	221, 4.7 (2.5)	58, 5.1 (2.4)	67, 4.4 (2.6)	67, 5.1 (2.4)	53, 5.7 (2.8)	146, 4.9 (2.4)	210, 4.9 (2.6)	25, 6.0 (2.5)	81, 5.9 (2.2)
2	186, 4.8 (2.5)	221, 4.4 (2.7)	52, 4.3 (2.6)	63, 4.1 (2.6)	62, 4.5 (3.0)	55, 4.9 (2.7)	144, 4.4 (2.4)	205, 4.6 (2.7)	26, 5.8 (2.6)	79, 5.6 (2.3)
3	192, 4.3 (2.7)	221, 4.1 (2.8)	52, 4.1 (2.8)	60, 3.9 (2.8)	62, 3.8 (2.9)	52, 4.8 (3.0)	145, 4.2 (2.6)	206, 4.6 (2.8)	27, 5.4 (2.4)	76, 5.8 (2.3)
4	180, 4.2 (2.7)	213, 4.2 (2.8)	53, 4.1 (2.7)	63, 3.8 (2.8)	62, 3.4 (2.8)	53, 4.6 (3.1)	136, 3.8 (2.5)	200, 4.5 (3.0)	24, 5.5 (2.8)	75, 5.7 (2.5)
5	176, 4.1 (2.9)	213, 4.2 (2.8)	50, 3.6 (2.7)	61, 3.9 (2.8)	62, 3.4 (2.7)	50, 4.4 (3.0)	137, 3.9 (2.6)	201, 4.3 (3.1)	26, 5.2 (2.9)	76, 5.4 (2.5)
6[$]	171, 4.0 (2.8)	214, 4.1 (2.9)	50, 3.5 (2.8)	61, 3.7 (2.8)	61, 3.6 (3.0)	48, 3.8 (2.8)	138, 3.6 (2.6)	198, 4.0 (3.0)	26, 5.2 (2.9)	72, 5.6 (2.6)
Average† (1-6 months)	205, 4.4 (2.3)	236, 4.3 (2.5)	61, 4.4 (2.4)	68, 3.9 (2.5)	67, 4.1 (2.5)	57, 4.8 (2.5)	154, 4.2 (2.2)	219, 4.6 (2.5)	28, 5.6 (2.4)	83, 5.6 (2.2)
Interaction										
[1]	-		.37 (-.50, 1.24) [P=0.40]		-.56 (-1.46, .34) [P=0.22]		-.48 (-1.10, .14) [P=0.13]		-.16 (-1.20, .89) [P=0.77]	
[2]	-.37 (-1.24, .50) [P=0.40]		-		-.93 (-2.03, .17) [P=.096]		-.85 (-1.75, .04) [P=.061]		-.53 (-1.75, .70) [P=0.40]	
[3]	.56 (-.34, -1.46) [P=0.22]		.93 (-.17, 2.03) [P=.096]		-		.08 (-.83, .99) [P=0.87]		.41 (-.84, 1.65) [P=0.52]	
[4]	.48 (-.14, 1.10) [P=0.13]		.85 (-.04, 1.75) [P=.061]		-.08 (-.99, .83) [P=0.87]		-		.33 (-.74, 1.39) [P=0.55]	

[Pre-specified reference site category = back] Between-group difference in mean scores (Stratified Care – Usual Care) by linear mixed model with practice and participants (random factors) and practice size and participants' age, gender, point-of-consultation pain score as well as interaction of pain site dummy variables x treatment arm (fixed factors).

[$] If the last monthly SMS/brief questionnaire response was missing it was imputed using the corresponding pain response from the returned 6-month questionnaire (if completed within 20 days of the date of issue).

† Average summary mean (SD) relate to the mean of available 1-6 month follow up data.

Interaction - reference site: [1] back; [2] neck; [3] shoulder; [4] knee.

Appendix 7: Screenshots of the risk-matched treatment options as presented to GPs (using example of a knee pain presentation)

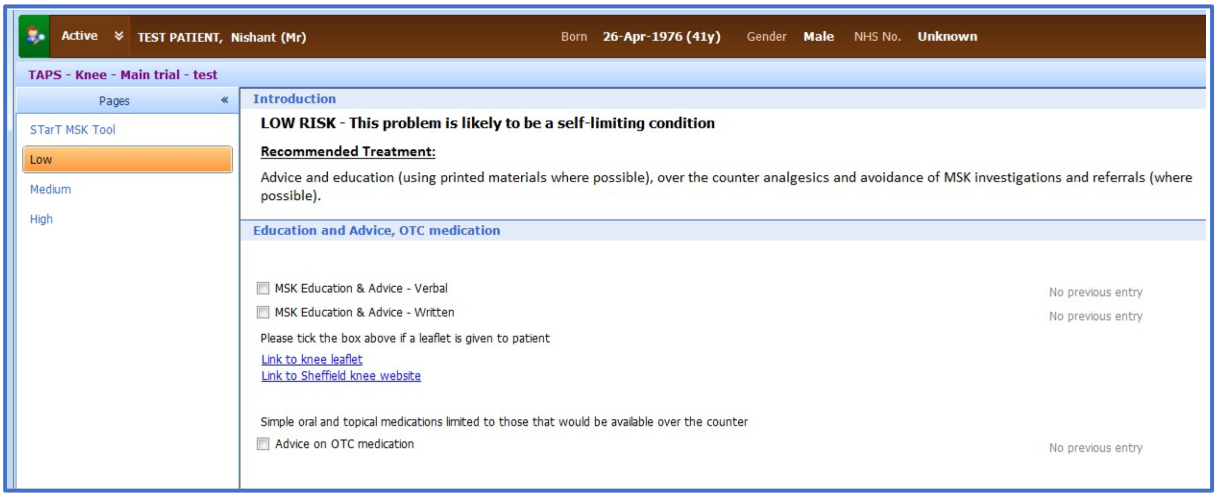

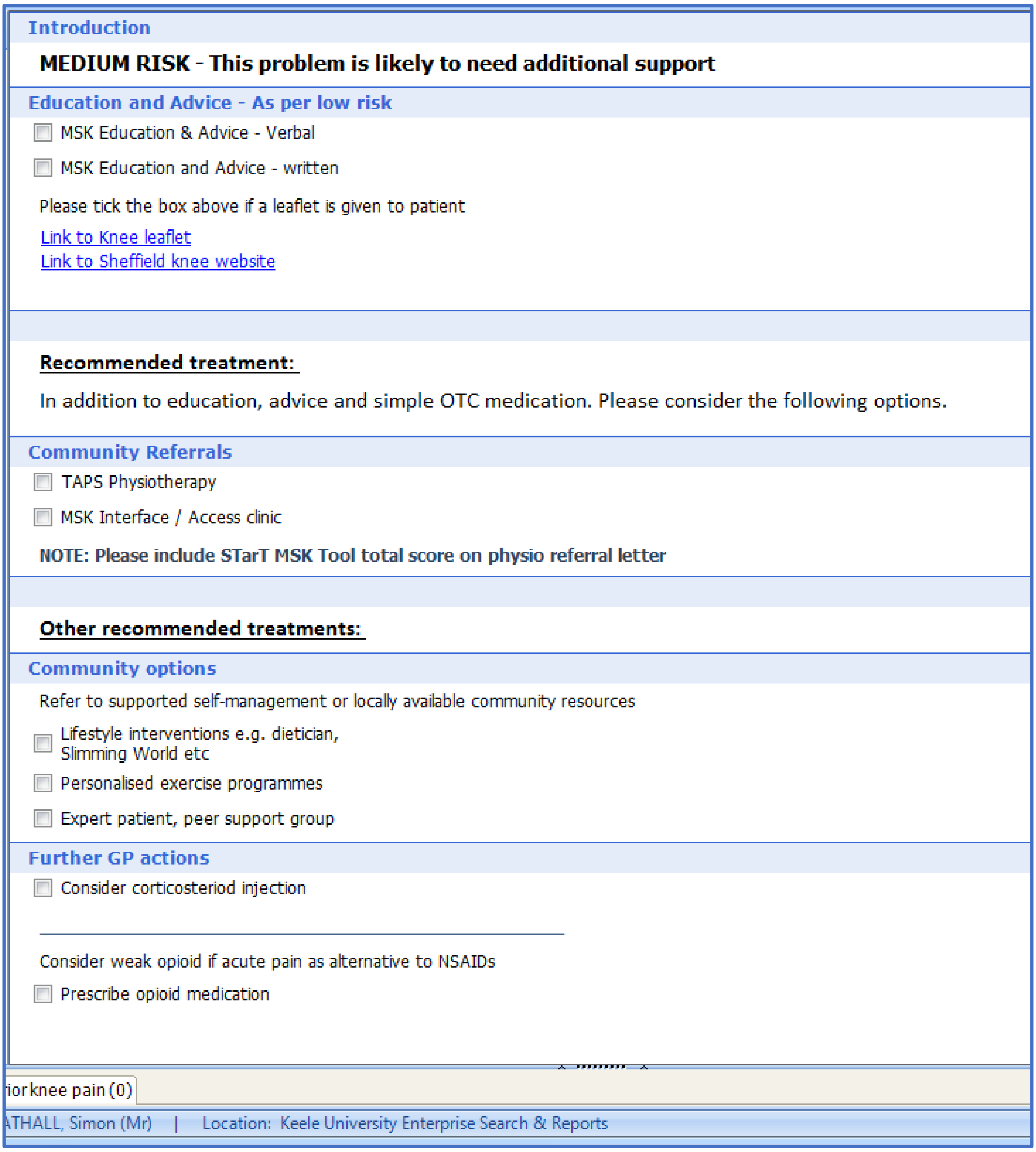

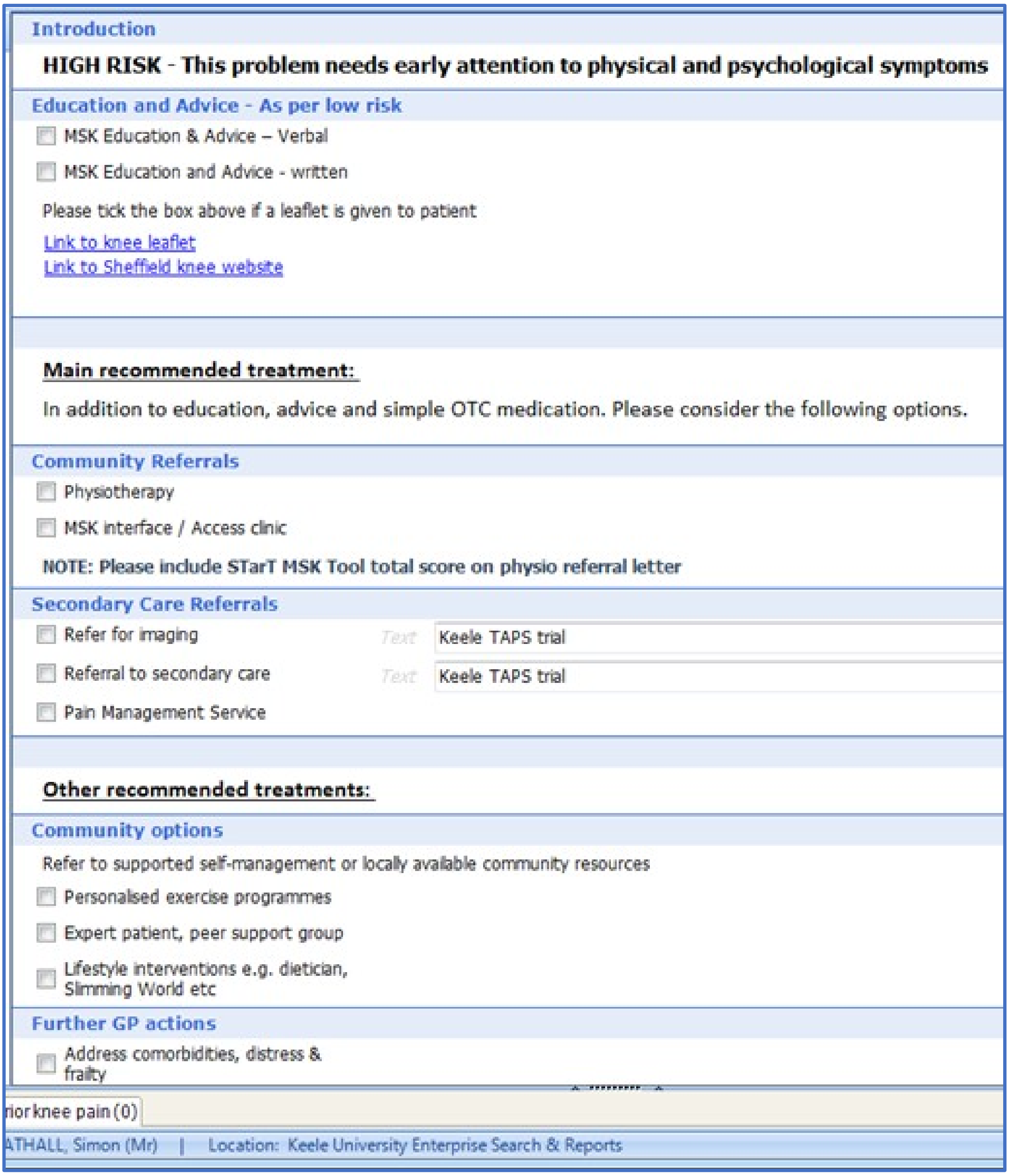

Introduction

HIGH RISK - This problem needs early attention to physical and psychological symptoms

Education and Advice - As per low risk

- [ ] MSK Education & Advice – Verbal
- [ ] MSK Education and Advice - written

Please tick the box above if a leaflet is given to patient

Link to knee leaflet
Link to Sheffield knee website

Main recommended treatment:

In addition to education, advice and simple OTC medication. Please consider the following options.

Community Referrals

- [ ] Physiotherapy
- [ ] MSK interface / Access clinic

NOTE: Please include STarT MSK Tool total score on physio referral letter

Secondary Care Referrals

- [ ] Refer for imaging — Text: Keele TAPS trial
- [ ] Referral to secondary care — Text: Keele TAPS trial
- [ ] Pain Management Service

Other recommended treatments:

Community options

Refer to supported self-management or locally available community resources

- [ ] Personalised exercise programmes
- [ ] Expert patient, peer support group
- [ ] Lifestyle interventions e.g. dietician, Slimming World etc

Further GP actions

- [ ] Address comorbidities, distress & frailty

rior knee pain (0)

ATHALL, Simon (Mr) | Location: Keele University Enterprise Search & Reports

Appendix 8: GP intervention fidelity showing the number and proportion of electronically tagged patients for whom the GP recorded the intention to provide a recommended matched treatment (Stratified Care arm) (based on the trial electronic medical record (EMR) template)

	Low Risk		Medium Risk		High Risk		Grand Total
	(n=227, 21.5%)		(n=585, 55.4%)		(n=244, 23.0%)		**1056**
Verbal advice given	161	71%	393	67%	143	59%	697
Written advice given	148	65%	338	58%	120	49%	606
Over-the-counter Medication	120	53%	13	2%	2	1%	135
MSK interface service referral	1	0%	73	12%	42	17%	116
Physiotherapy referral	22	10%	378	65%	147	60%	547
Exercise programme	0	0%	56	10%	30	12%	86
Expert peer support	0	0%	26	4%	23	9%	49
Lifestyle advice/intervention	1	0%	52	9%	32	13%	85
Opioid medication	6	3%	135	23%	80	33%	221
Corticosteroid injection	1	0%	19	3%	11	5%	31
Pain Management service referral	0	0%	0	0%	7	3%	7
Referral to secondary care	5	2%	37	6%	32	13%	74
Referral for imaging	9	4%	20	3%	71	29%	100
Prescribed atypical analgesia	0	0%	2	0%	49	20%	51
Addressed comorbidities	0	0%	0	0%	28	12%	28
	474		1542		817		2833

Number and proportion of invited patients for whom the GP recorded selection of a recommended matched treatment (based on GP entered information onto the trial specific EMR template)	**n**	**% of patient risk subgroup**	**% of total**
Per protocol (Overall)	815	-	77.2
Low risk - per protocol	176	77.5	16.7
Medium risk - per protocol	457	78.1	43.3
High risk - per protocol	182	74.9	17.2
Treatment not in line with the stratified care protocol			
Low risk - given treatments for patients at Medium risk	17	7.5	1.6
Low risk - given treatments for patients at High risk	9	4.0	0.9
Low risk – only tool used (no treatments selected)	27	11.9	2.6
Medium risk – given treatments for patients at Low risk	66	11.3	6.3
Medium risk - given treatments for patients at High risk	15	2.6	1.4
Medium risk – only tool used (no treatments selected)	50	8.5	4.7
High risk – given treatments for patients at Low risk	2	0.8	0.2
High risk – given treatments for patients at Medium risk	27	11.1	2.6
High risk – only tool used (no treatments selected)	27	11.1	2.6
Incomplete tool	1	-	0.1
Grand Total	1056		

Appendix 9: Overall and Monthly Pain Intensity (NRS) scores per patient risk subgroup

	Low risk		Medium risk		High risk	
**Month**	Stratified Care	Usual Care	Stratified Care	Usual Care	Stratified Care	Usual Care
Clinical version [n, mean (SD)]						
1	93, 2.8 (2.0)	123, 2.6 (2.1)	218, 5.2 (2.0)	267, 5.0 (2.2)	156, 6.3 (2.3)	199, 6.4 (2.3)
2	88, 2.5 (2.0)	119, 2.4 (2.2)	209, 4.7 (2.3)	262, 4.6 (2.3)	150, 5.7 (2.5)	199, 5.9 (2.6)
3	88, 2.4 (2.0)	120, 2.4 (2.3)	216, 4.2 (2.4)	258, 4.4 (2.6)	151, 5.3 (2.8)	195, 5.9 (2.7)
4	88, 2.2 (1.9)	116, 2.4 (2.3)	205, 4.0 (2.6)	259, 4.4 (2.7)	139, 5.1 (2.7)	188, 5.8 (2.7)
5	87, 2.0 (2.1)	117, 2.2 (2.3)	201, 3.9 (2.5)	252, 4.2 (2.7)	142, 5.0 (2.9)	192, 5.7 (2.7)
6$	85, 1.9 (2.1)	114, 2.0 (2.1)	198, 3.8 (2.6)	249, 4.0 (2.7)	143, 4.9 (2.9)	191, 5.5 (2.8)
Average† (1-6 months)	93, 2.3 (1.7)	124, 2.3 (1.9)	233, 4.4 (2.0)	282, 4.4 (2.1)	164, 5.4 (2.2)	210, 5.9 (2.3)
Interaction#			-.01 (-.64, .62) [P=.98]		-.30 (-.97, .36) [P=.37]	

Between-group difference in mean scores (Stratified Care – Usual Care) by linear mixed model with practice and participants (random factors) and practice size and participants' age, gender, point-of-consultation pain score, time as well as interaction of STarT MSK Tool clinical subgroup dummy variables (Low risk subgroup = reference) x treatment arm (fixed factors).

$ If the last monthly SMS/brief questionnaire response was missing it was imputed using the corresponding pain response from the returned 6-month questionnaire (if completed within 20 days of the date of issue).

† Average summary mean (SD) relate to the mean of available 1-6 month follow up data.

Appendix 10: Summary of overall and monthly NRS-psychological distress and NRS-pain self-efficacy (secondary outcome measures)

Month	n_{SC}, n_{UC}	Stratified Care mean (SD)	Usual Care mean (SD)	mean difference# (95% CI)	SMD (95% CI)	P value
Psychological distress						
1	489, 625	4.3 (2.7)	4.3 (2.7)	0.05 (-0.37, 0.47)	0.02 (-0.14, 0.18)	0.82
2	468, 615	4.0 (2.8)	4.0 (2.8)	0.11 (-0.32, 0.53)	0.04 (-0.12, 0.20)	0.63
3	472, 610	3.8 (2.8)	4.0 (2.9)	-0.20 (-0.63, 0.23)	-0.08 (-0.24, 0.09)	0.37
4	453, 595	3.5 (2.8)	4.0 (3.0)	-0.44 (-0.88, -0.01)	-0.17 (-0.34, -0.002)	0.047
5	448, 598	3.5 (2.8)	3.9 (3.0)	-0.38 (-0.82, 0.07)	-0.15 (-0.32, 0.03)	0.098
6$	427, 573	3.4 (2.8)	3.8 (2.9)	-0.25 (-0.71, 0.22)	-0.10 (-0.27, 0.08)	0.30
Average#	514, 663	3.8 (2.4)	4.0 (2.5)	-0.19 (-0.58, 0.21)	-0.07 (-0.23, 0.08)	0.36
Pain self-efficacy						
1	484, 621	6.2 (2.5)	6.2 (2.7)	0.05 (-0.30, 0.40)	0.02 (-0.12, 0.16)	0.78
2	463, 609	6.4 (2.7)	6.5 (2.7)	-0.12 (-0.47, 0.23)	-0.05 (-0.19, 0.09)	0.51
3	468, 609	6.5 (2.7)	6.5 (2.8)	0.01 (-0.34, 0.37)	0.005 (-0.14, 0.15)	0.94
4	450, 591	6.4 (3.0)	6.5 (2.8)	-0.02 (-0.39, 0.35)	-0.009 (-0.15, 0.14)	0.90
5	445, 596	6.5 (2.9)	6.6 (2.8)	-0.02 (-0.40, 0.36)	-0.008 (-0.16, 0.14)	0.92
6$	429, 565	6.5 (3.0)	6.6 (2.8)	-0.001 (-0.39, 0.39)	-0.006 (-0.15, 0.15)	0.99
Average#	515, 663	6.3 (2.2)	6.4 (2.1)	-0.02 (-0.31, 0.27)	-0.007 (-0.12, 0.11)	0.91

SC = Stratified Care; UC = Usual Care. n_{SC} = number of data analysed in the SC arm; n_{UC} = number of data analysed in the UC arm.

Between-group difference in mean scores (Usual Care – Stratified Care) by linear mixed model with Practice and Participants (random factors) and practice size and participants' age, gender and (point-of-consultation) pain score and corresponding baseline questionnaire psychological distress or pain self-efficacy score (fixed factors). Average = mean of available 1-6 month follow up data.

$ If the last monthly SMS/brief questionnaire response is missing it will be imputed using the corresponding psychological distress / pain self-efficacy response from the returned 6-month questionnaire (if available and completed within 20 days of the date of issue of their 6-month SMS/brief questionnaire)

Appendix 11: Sensitivity analyses reported for the primary outcome analysis

Outcomes	Mean difference[1]/OR[2] (95% CI)	P-value
Mean differences[1]		
**1-6 months**		
Constrained baseline model	-0.18 (-0.53, 0.16)	0.30
Per protocol analysis (based on consultation template)	-0.11 (-0.62, 0.41)	0.68
Complier Average Casual Effect analysis (compliance based on consultation template)	0.47 (-2.16, 3.10)	0.73
Multiple Imputation (inclusive of additional baseline covariates)	-0.03 (-0.48, 0.42)	0.90
Practice-level analysis	-0.29 (-0.95, 0.36)	0.36
**4-6 months**		
Constrained baseline model	-0.35 (-0.74, 0.04)	0.082
Per protocol analysis (based on consultation template)	-0.30 (-0.84, 0.23)	0.27
Complier Average Casual Effect analysis (compliance based on consultation template)	0.93 (-1.98, 3.83)	0.53
Multiple Imputation (inclusive of additional baseline covariates)	-0.15 (-0.64, 0.33)	0.54
Practice-level analysis	-0.45 (-1.16, 0.27)	0.21
**With/without complete monthly follow-up**		
Participants with 6/6 (100%) monthly follow-up responses	-0.24 (-0.76, 0.39)	0.39
Participants with incomplete (<6) monthly follow-up responses	0.01 (0.68, -0.65)	0.97
Odds Ratios[2]		
**1-6 months**		
MCIC 2-point change	1.34 (0.75, 2.42)	0.32
MCIC 3-point change	1.28 (0.71, 2.32)	0.41
**4-6 months**		
MCIC 2-point change	1.87 (1.01, 3.43)	0.045
MCIC 3-point change	1.88 (1.02, 3.48)	0.044

[1] Between-group difference in mean scores (Stratified Care – Usual Care) by linear mixed model with practice and participants (random factors) and practice size and participants' age, gender and (point-of-consultation) pain score (fixed factors).

[2] Between-group comparison (odds ratio of Stratified Care to Usual Care) by generalised mixed model (logit link function) of long-dataset with practice and participants (random factors) and practice size and participants' age, gender and (point-of-consultation) pain score (fixed factors).

Appendix 12: Sensitivity analysis looking at primary outcome analysis (NRS pain) split by Females and Males (separate analyses)

Month	n_{SC}, n_{UC}	Stratified Care, mean (SD)	Usual Care, mean (SD)	Mean difference (95% CI)[#]	P-value
Females					
PoC	313, 400	7.1 (1.8)	6.9 (1.9)	-	-
Baseline	312, 399	6.6 (2.2)	6.5 (2.2)	-	-
1	289, 380	5.4 (2.4)	5.1 (2.5)	0.38 (-0.21, 0.96)	0.20
2	277, 366	5.0 (2.6)	4.7 (2.7)	0.36 (-0.22, 0.95)	0.23
3	280, 367	4.6 (2.8)	4.6 (2.8)	0.04 (-0.55, 0.64)	0.89
4	265, 357	4.4 (2.6)	4.6 (2.8)	-0.13 (-0.73, 0.48)	0.68
5	264, 355	2.8 (4.0)	4.4 (2.9)	0.05 (-0.56, 0.67)	0.87
6[$]	259, 348	4.2 (2.8)	4.2 (2.9)	0.11 (-0.52, 0.74)	0.75
Average (1-6 months)[†]	302, 395	4.7 (2.3)	4.6 (2.4)	0.14 (-0.42, 0.69)	0.63
Average (4-6 months)	283, 383	4.4 (2.5)	4.4 (2.7)	0.07 (-0.54, 0.68)	0.83
Males					
PoC	221, 277	6.4 (1.9)	6.4 (2.1)	-	-
Baseline	221, 276	6.0 (2.2)	6.2 (2.3)	-	-
1	202, 252	4.7 (2.5)	4.9 (2.7)	-0.22 (-0.86, 0.41)	0.49
2	193, 257	4.2 (2.5)	4.6 (2.7)	-0.41 (-1.04, 0.23)	0.21
3	198, 248	3.7 (2.5)	4.4 (2.9)	-0.67 (-1.31, 0.03)	0.040
4	190, 247	3.6 (2.8)	4.4 (3.0)	-0.88 (-1.53, -0.22)	0.009
5	187, 246	3.3 (2.6)	4.3 (3.0)	-1.02 (-1.68, 0.35)	0.003
6[$]	187, 245	3.3 (2.7)	4.1 (2.9)	-0.83 (-1.51, -0.14)	0.018
Average (1-6 months)[†]	213, 268	3.8 (2.3)	4.5 (2.6)	-0.67 (-1.27, -0.07)	0.028
Average (4-6 months)	203, 259	3.5 (2.5)	4.3 (2.8)	-0.79 (-1.49, -0.09)	0.027

n_{SC} = number of data analysed in the Stratified Care arm; n_{UC} = number of data analysed in the Usual Care arm.

[#] Between-group difference in mean scores (Stratified Care – Usual Care) by linear mixed model with practice and participants (random factors) and practice size and participants' age, gender and (point-of-consultation) pain score (fixed factors).

[$] If the last monthly SMS/brief questionnaire response was missing it was imputed using the corresponding pain response from the returned 6-month questionnaire (if completed within 20 days of the date of issue).

[†] Primary endpoint (average of available data for 1-6 months follow up). Average summary mean (SD) relates to the mean of available 1-6 month follow up data.

Appendix 13: Comparison of GP decision-making per arm (0-182 days) taken from the anonymised electronic medical record audit

	**Anonymised EMR audit data on all patients with a completed trial recruitment template (n=2494)**			**Trial participants with known baseline risk subgroup taken from the baseline questionnaire (n=1130)**					
	**Both Usual Care and Stratified Care**	**Usual Care practices**	**Stratified Care practices**	**Low risk† (n=224, 20%)**		**Medium risk† (n=524, 46%)**		**High risk† (n=382, 34%)**	
	All patients (n=2494)	All patients (n=1438)	All patients (n=1056)	Usual Care (n=126)	Stratified Care (n=98)	Usual Care (n=286)	Stratified Care (n=238)	Usual Care (n=215)	Stratified Care (n=167)
**Prescription**, count (mean per patient)									
Simple analgesics	For use								
Overall 0-182 days	1375 (0.55)	861 (0.60)	514 (0.49)	**19 (0.15)**	**38 (0.39)**	117 (0.41)	136 (0.57)	282 (1.31)	165 (0.99)
0-7 days (PoC)	312 (0.13)	**116 (0.08)**	**196 (0.19)**	**5 (0.04)**	**34 (0.35)**	**12 (0.04)**	**45 (0.19)**	39 (0.18)	22 (0.13)
N (%) ≥1 in 0-7 days (PoC)	266 (10.7%)	**90 (6.3%)**	**176 (16.7%)**	**5 (4.0%)**	**32 (32.7%)**	**11 (3.8%)**	**37 (15.5%)**	28 (13.0%)	17 (10.2%)
8-182 days	1063 (0.43)	745 (0.52)	318 (0.30)	14 (0.11)	4 (0.04)	105 (0.37)	91 (0.38)	243 (1.13)	143 (0.86)
Anti-inflammatories									
Overall 0-182 days	1965 (0.79)	1145 (0.80)	820 (0.78)	52 (0.41)	35 (0.36)	212 (0.74)	138 (0.58)	286 (1.33)	232 (1.39)
0-7 days (PoC)	580 (0.23)	331 (0.23)	249 (0.24)	19 (0.15)	21 (0.21)	54 (0.19)	45 (0.19)	63 (0.29)	36 (0.22)
N (%) ≥1 in 0-7 days (PoC)	511 (20.5%)	300 (20.9%)	211 (20.0%)	18 (14.3%)	17 (17.3%)	51 (17.8%)	41 (17.2%)	51 (23.7%)	28 (16.8%)
8-182 days	1385 (0.56)	814 (0.57)	571 (0.54)	33 (0.26)	14 (0.14)	158 (0.55)	93 (0.39)	223 (1.04)	196 (1.17)
Neuromodulators									
Overall 0-182 days	2929 (1.17)	1859 (1.29)	1070 (1.01)	51 (0.40)	31 (0.32)	244 (0.85)	238 (1.00)	619 (2.88)	376 (2.25)
0-7 days (PoC)	378 (0.15)	218 (0.15)	160 (0.15)	7 (0.06)	8 (0.08)	37 (0.13)	26 (0.11)	54 (0.25)	45 (0.27)
N (%) ≥1 in 0-7 days (PoC)	269 (10.8%)	159 (11.1%)	110 (10.4%)	5 (4.0%)	7 (7.1%)	26 (9.1%)	16 (6.7%)	37 (17.2%)	30 (18.0%)
8-182 days	2551 (1.02)	1641 (1.14)	910 (0.86)	44 (0.35)	23 (0.23)	207 (0.72)	153 (0.64)	565 (2.63)	331 (1.98)
Muscle relaxants									
Overall 0-182 days	667 (0.27)	**452 (0.31)**	**215 (0.20)**	6 (0.05)	6 (0.06)	46 (0.16)	32 (0.13)	61 (0.28)	49 (0.29)
0-7 days (PoC)	191 (0.08)	123 (0.09)	68 (0.06)	3 (0.02)	5 (0.05)	20 (0.07)	12 (0.05)	16 (0.07)	13 (0.08)

N (%) ≥1 in 0-7 days (PoC)	164 (6.6%)	101 (7.0%)	63 (6.0%)	3 (2.4%)	5 (5.1%)	19 (6.6%)	12 (5.0%)	16 (7.4%)	12 (7.2%)
8-182 days	476 (0.19)	329 (0.23)	147 (0.14)	3 (0.02)	1 (0.01)	26 (0.09)	20 (0.08)	45 (0.21)	36 (0.22)
Weak opioids									
Overall 0-182 days	3803 (1.52)	2241 (1.56)	1562 (1.48)	69 (0.55)	33 (0.34)	333 (1.16)	305 (1.28)	588 (2.73)	414 (2.48)
0-7 days (PoC)	1035 (0.41)	**458 (0.32)**	**577 (0.55)**	**13 (0.10)**	**25 (0.26)**	**79 (0.28)**	**111 (0.47)**	**93 (0.43)**	**111 (0.66)**
N (%) ≥1 in 0-7 days (PoC)	714 (28.6%)	376 (26.1%)	338 (32.0%)	13 (10.3%)	16 (16.3%)	69 (24.1%)	71 (29.8%)	70 (32.6%)	65 (38.9%)
8-182 days	2768 (1.11)	1783 (1.24)	985 (0.93)	56 (0.44)	8 (0.08)	254 (0.89)	194 (0.82)	495 (2.30)	303 (1.81)
Long term prescribing^, n (%)	308 (12.3%)	179 (12.4%)	129 (12.2%)	3 (2.4%)	4 (4.1%)	38 (13.3%)	30 (12.6%)	42 (19.5%)	32 (19.2%)
Strong opioids									
Overall 0-182 days	595 (0.24)	244 (0.17)	351 (0.33)	0 (0.00)	11 (0.11)	**18 (0.06)**	**55 (0.23)**	89 (0.41)	62 (0.37)
0-7 days (PoC)	250 (0.10)	**24 (0.02)**	**226 (0.21)**	0 (0.00)	11 (0.11)	0 (0.00)	40 (0.17)	**7 (0.03)**	**47 (0.28)**
N (%) ≥1 in 0-7 days (PoC)	228 (9.1%)	**14 (1.0%)**	**214 (20.3%)**	0 (0%)	11 (11.2%)	0 (0%)	40 (16.8%)	**6 (1.0%)**	**43 (25.7%)**
8-182 days	345 (0.14)	220 (0.15)	125 (0.12)	0 (0.00)	0 (0.00)	18 (0.06)	15 (0.06)	82 (0.38)	15 (0.09)
Long term prescribing^, n (%)	26 (1.0%)	13 (0.9%)	13 (1.2%)	0 (0%)	0 (0%)	0 (0%)	1 (0.4%)	6 (2.8%)	4 (2.4%)
Corticosteroid injection									
Overall 0-182 days	244 (0.10)	155 (0.11)	89 (0.08)	13 (0.10)	8 (0.08)	36 (0.13)	31 (0.13)	39 (0.18)	18 (0.11)
0-7 days (PoC)	83 (0.03)	39 (0.03)	44 (0.04)	4 (0.03)	5 (0.05)	8 (0.03)	20 (0.08)	7 (0.03)	6 (0.04)
N (%) ≥1 in 0-7 days (PoC)	72 (2.9%)	39 (2.7%)	33 (3.1%)	4 (3.2%)	3 (3.1%)	8 (2.8%)	14 (5.9%)	7 (3.3%)	5 (3.0%)
8-182 days	161 (0.06)	116 (0.08)	45 (0.04)	9 (0.07)	3 (0.03)	28 (0.10)	11 (0.05)	32 (0.15)	12 (0.07)
**Referral**, count (mean per patient)									
Physiotherapy or Musculoskeletal interface clinic									
Overall 0-182 days	1176 (0.47)	**303 (0.21)**	**873 (0.83)**	**26 (0.21)**	**60 (0.61)**	**73 (0.26)**	**230 (0.97)**	**43 (0.2)**	**164 (0.98)**
0-7 days (PoC)	815 (0.33)	**143 (0.10)**	**672 (0.64)**	**15 (0.12)**	**41 (0.42)**	**32 (0.11)**	**166 (0.70)**	**18 (0.08)**	**127 (0.76)**
N (%) ≥1 in 0-7 days (PoC)	810 (32.5%)	**142 (9.9%)**	**668 (63.3%)**	**15 (11.9%)**	**41 (41.8%)**	**32 (11.2%)**	**166 (69.7%)**	**18 (8.4%)**	**126 (75.4%)**
8-182 days	361 (0.14)	160 (0.11)	201 (0.19)	11 (0.09)	2 (0.02)	41 (0.14)	64 (0.27)	25 (0.12)	37 (0.22)
Specialist orthopaedics									
Overall 0-182 days	575 (0.23)	357 (0.25)	218 (0.21)	24 (0.19)	11 (0.11)	65 (0.23)	52 (0.22)	72 (0.33)	44 (0.26)

0-7 days (PoC)	184 (0.07)	120 (0.08)	64 (0.06)	8 (0.06)	4 (0.04)	19 (0.07)	11 (0.05)	26 (0.12)	11 (0.07)
N (%) ≥1 in 0-7 days (PoC)	176 (7.1%)	112 (7.8%)	64 (6.1%)	7 (5.6%)	4 (4.1%)	18 (6.3%)	11 (4.6%)	22 (10.2%)	11 (6.6%)
8-182 days	391 (0.16)	237 (0.16)	154 (0.15)	16 (0.13)	7 (0.07)	46 (0.16)	41 (0.17)	46 (0.21)	33 (0.20)
Pain clinic									
Overall 0-182 days	43 (0.02)	36 (0.03)	7 (0.01)	0 (0.00)	0 (0.00)	3 (0.01)	1 (0.004)	8 (0.04)	4 (0.02)
0-7 days (PoC)	13 (0.01)	12 (0.008)	1 (0.001)	0 (0.00)	0 (0.00)	1 (0.003)	0 (0.00)	3 (0.01)	1 (0.006)
N (%) ≥1 in 0-7 days (PoC)	13 (0.5%)	12 (0.8%)	1 (0.1%)	0 (0%)	0 (0%)	1 (0.3%)	0 (0%)	3 (1.4%)	1 (0.6%)
8-182 days	30 (0.01)	24 (0.02)	6 (0.01)	0 (0.00)	0 (0.00)	2 (0.01)	1 (0.004)	5 (0.02)	3 (0.02)
Rheumatology									
Overall 0-182 days	42 (0.02)	25 (0.02)	17 (0.02)	1 (0.01)	0 (0.00)	7 (0.02)	3 (0.01)	9 (0.04)	2 (0.01)
0-7 days (PoC)	7 (0.003)	4 (0.003)	3 (0.003)	0 (0.00)	0 (0.00)	2 (0.007)	1 (0.004)	2 (0.01)	0 (0.00)
N (%) ≥1 in 0-7 days (PoC)	7 (0.3%)	4 (0.3%)	3 (0.3%)	0 (0%)	0 (0%)	2 (0.7%)	1 (0.4%)	2 (0.9%)	0 (0%)
8-182 days	35 (0.01)	21 (0.02)	14 (0.01)	1 (0.008)	0 (0.00)	5 (0.02)	2 (0.01)	7 (0.03)	2 (0.01)
**Imaging**, count (mean per patient)									
X-ray or MRI for musculoskeletal disorder									
Overall 0-182 days	604 (0.24)	468 (0.33)	136 (0.13)	**28 (0.22)**	**3 (0.03)**	84 (0.29)	33 (0.14)	82 (0.38)	26 (0.16)
0-7 days (PoC)	125 (0.05)	99 (0.07)	26 (0.02)	10 (0.08)	1 (0.01)	22 (0.08)	8 (0.03)	18 (0.08)	2 (0.01)
N (%) ≥1 in 0-7 days (PoC)	96 (3.8%)	75 (5.2%)	21 (2.0%)	8 (6.3%)	1 (1.0%)	15 (5.2%)	6 (2.5%)	15 (7.0%)	2 (1.2%)
8-182 days	479 (0.19)	369 (0.26)	110 (0.10)	18 (0.14)	2 (0.02)	62 (0.22)	25 (0.11)	64 (0.30)	24 (0.14)
Ultrasound scan for musculoskeletal disorder									
Overall 0-182 days	97 (0.04)	70 (0.05)	27 (0.03)	5 (0.04)	1 (0.01)	9 (0.03)	8 (0.03)	10 (0.05)	6 (0.04)
0-7 days (PoC)	2 (0.001)	1 (0.001)	1 (0.001)	1 (0.008)	0 (0.00)	0 (0.00)	0 (0.00)	0 (0.00)	0 (0.00)
N (%) ≥1 in 0-7 days (PoC)	2 (0.1%)	1 (0.1%)	1 (0.1%)	1 (0.8%)	0 (0%)	0 (0%)	0 (0%)	0 (0%)	0 (0%)
8-182 days	95 (0.04)	69 (0.01)	26 (0.03)	4 (0.03)	1 (0.01)	9 (0.03)	8 (0.03)	10 (0.05)	6 (0.04)
Bone density scan									
Overall 0-182 days	63 (0.03)	46 (0.03)	17 (0.02)	0 (0.00)	2 (0.02)	9 (0.03)	2 (0.01)	12 (0.06)	10 (0.06)
0-7 days (PoC)	1 (0.001)	1 (0.001)	0 (0)	0 (0.00)	0 (0.00)	1 (0.003)	0 (0.00)	0 (0.00)	0 (0.00)
N (%) ≥1 in 0-7 days (PoC)	1 (0.1%)	1 (0.1%)	0 (0%)	0 (0%)	0 (0%)	1 (0.3%)	0 (0%)	0 (0%)	0 (0%)

8-182 days	62 (0.03)	45 (0.03)	17 (0.02)	0 (0.00)	2 (0.02)	8 (0.03)	2 (0.01)	12 (0.06)	10 (0.06)
**Sick certification,** count (mean per patient)									
Overall 0-182 days	1181 (0.47)	728 (0.51)	453 (0.43)	**33 (0.26)**	**6 (0.06)**	69 (0.24)	52 (0.22)	154 (0.72)	187 (1.12)
0-7 days (PoC)	325 (0.13)	205 (0.14)	120 (0.11)	17 (0.13)	5 (0.05)	16 (0.06)	26 (0.11)	27 (0.13)	24 (0.14)
N (%) ≥1 in 0-7 days (PoC)	213 (8.5%)	128 (8.9%)	85 (8.0%)	7 (5.6%)	4 (4.1%)	14 (4.9%)	16 (6.7%)	16 (7.4%)	15 (9.0%)
8-182 days	1033 (0.41)	598 (0.42)	435 (0.41)	16 (0.13)	1 (0.01)	53 (0.19)	26 (0.11)	127 (0.59)	163 (0.98)
**Repeat Musculoskeletal GP consultations over 6 months**									
(8-182 days), count (mean per patient)	2404 (0.96)	1389 (0.97)	1015 (0.96)	51 (0.40)	32 (0.33)	233 (0.81)	217 (0.91)	383 (1.78)	284 (1.7)

† STarT MSK tool scored as 0-4= low-risk; 5-8= medium risk; 9-12= high risk.
^ Long term prescribing was calculated as the number of patients who received at least one prescription in both the 0-7 days period, and the 8-182 days period.

Statistical testing of these process measures was post-hoc analyses with no adjustment for multiple testing due to their exploratory nature. Analyses were carried out by negative binomial mixed model with practice (random factor) and practice size and participants' point-of-consultation pain score (fixed factors) for count data, except where logistic mixed modelling with the same fixed/random factors was used instead (for binary data, or due to lack of model convergence and/or small counts). Ratios are for Stratified Care relative to Usual Care. Statistically significant p-values (p<0.05) are bolded.

Receipt of written information by the GP was also asked in the baseline patient questionnaire;
Overall, number (%) of 'Yes' responses = **306/528 (58.0%)** in Stratified Care and **175/672 (26.0%)** in Usual Care **(*p*<0.0001)**
by risk subgroup:
- Low risk subgroup = 65/98 (66.3%) in Stratified Care and 31/126 (24.6%) in Usual Care
- Medium risk subgroup = 139/236 (58.9%) in Stratified Care and 79/283 (27.9%) in Usual Care
- High risk subgroup = 84/164 (51.2%) in Stratified Care and 53/213 (24.9%) in Usual Care

DATA ANALYSIS PLAN (for the STarT MSK Trial Protocol)
[Patient facing name: The TAPS study] Treatment for Aches and PainS

Trial registration number: ISRCTN15366334

Study team:

Chief Investigator
Professor Nadine Foster, NIHR Professor of Musculoskeletal Health in Primary Care, (1) Telephone: 01782 734705, email: n.foster@keele.ac.uk

Principal Investigator - Keele
Dr Jonathan Hill, Senior Lecturer in Physiotherapy, (1) Telephone: 01782 733900, email: j.hill@keele.ac.uk

Trial Management is coordinated by Keele Clinical Trials Unit (CTU)

Trial Manager: Ms Stefannie Garvin, (3) Telephone: 01782 733964, email: s.garvin@keele.ac.uk
Study Coordinator: Dr Andrea Cherrington,(3) Telephone 01782 734875, email: a.cherrington@keele.ac.uk
Trial Administrator: Ms Alicia Bratt, (3) Telephone: 01782 733921, email: a.j.bratt@keele.ac.uk
Health Informatics: Mr Simon Wathall, (3) Telephone: 01782 734856, email: s.wathall@keele.ac.uk

Statistics
Dr Martyn Lewis, Reader in Biostatistics, (1) Telephone: 01782 734849, email: a.m.lewis@keele.ac.uk
Dr Ying Chen, Research Associate in Biostatistics, (1) Telephone: 01782 733990, email: y.chen1@keele.ac.uk
Mr Kieran Bromley, Research Assistant in Biostatistics, (1) Telephone 01782 733926, email: k.bromley1@keele.ac.uk
Health Economics
Dr Sue Jowett, Reader in Health Economics, Health Economics Unit, (2) Telephone 0121 4147898, email: s.jowett@bham.ac.uk
Dr Ray Oppong, Lecturer in Health Economics, Health Economics Unit, (2) Telephone 0121 4147065, email: r.a.oppong@bham.ac.uk
Dr Jesse Kigozi, Research Fellow in Health Economics, Health Economics Unit, (2) Telephone 0121 414 8971, email: j.kigozi@bham.ac.uk

Qualitative researchers
Dr Ben Saunders, Research Associate: Health Services Research, (1) Telephone: 01782 734880, email: b.saunders@keele.ac.uk

Sponsor's Representative
Dr Tracy Nevatte, Head of Project Assurance, Legal & Compliance Team (1) Telephone: 01782 734714, email: t.nevatte@keele.ac.uk.

(1) = Primary Care Centre Versus Arthritis / (2) = Health Economics Unit, Birmingham University / (3) = Keele CTU

Trial Management Group

Professor Nadine Foster (CI), Dr Jonathan Hill (Research PI and TMG Chair), Professor Jo Protheroe (Academic GP), Professor Kate Dunn (Epidemiologist), Dr Martyn Lewis (Senior Statistician), Dr Ben Saunders (Qualitative researcher), Ms Hollie Birkinshaw (Qualitative researcher), Mr Simon Wathall (Health Informatics Specialist), Ms Stefannie Garvin (Trial Manager), Dr Adrian Chudyk (Clinical Lecturer), Dr Vincent Cooper (Senior Lecturer in General Practice), .

Clinician Training and Support Group

GPs - Dr Vince Cooper, Dr Adrian Chudyk

Physiotherapists – Dr Jonathan Hill, Ms Gail Sowden and Ms Kay Stevenson.

Specialist Advisers

Ms Adele Higginbottom and Ms Carol Rhodes (Patient and Public Involvement & Engagement), Dr Sue Jowett and Dr Ray Oppong (Health Economists), Mrs Jenny Stevens (CRN), Mrs Helen Duffy (SLA's and Consortium), Professor Kate Dunn (WP1 lead), Mr John Murphy (PPIE Perspectives), Professor Carolyn Chew-Graham, (Professor of General Practice Research), Professor Elaine Hay (Professor of Community Rheumatology), Professor Christian Mallen (NIHR Professor, General Practice Research), and Professor Danielle van der Windt (Professor, Primary Care Epidemiology).

Trial Steering Committee (TSC)

Prof Tom Fahey (Chair: Professor of General Practice, Royal College of Surgeons, Ireland), Dr Caroline Sanders (Reader in Medical Sociology, University of Manchester), Dr Richard Stevens (Medical Statistics Lecturer, Oxford University), Mr John Murphy and Mrs Angie Emery (PPIE perspectives).

Data Monitoring Committee (DMC)

Dr Ricky Mullis, Independent member (Chair) and Senior Research Associate at the General Practice and Primary Care Research Unit, University of Cambridge; Dr Nicola Walsh, Independent member and Associate Professor of Musculoskeletal Rehabilitation, University of West England; Dr Terrence O'Neill, Independent member and Professor of Rheumatology and Clinical Epidemiology, University of Manchester; Dr Karla Hemming, Independent member and Senior Lecturer in Biostatistics, University of Birmingham.

Authors of the DAP:

Senior Statistician .... Signature (Date) ...30/01/2020

Trial Statistician .... Signature (Date) 30/01/2020

Senior Health Economist... Signature (Date) 29/01/2020

Trial Economist Signature (Date) 29/01/2020

Trial Economist Signature (Date) 29/01/2020

Principal Academic Investigator ...Signature (Date) ...3/2/2020...

Chief Investigator.......................... Signature (Date) ...7/2/2020

Version 1.0 Date: 28th January 2020

Declaration regarding undertaking of the analysis

Mr Kieran Bromley (statistician) will be undertaking data cleaning and analysis of the study data under the guidance and supervision of Dr Martyn Lewis (senior statistician) who will double-analyse primary and key secondary outcomes.

With reference to the main trial protocol version: 1.2, 19th July 2018

Version History Log

This section should detail the key elements of the changes to the successive versions.

Version	Date implemented	Section no. changed	Details of changes (to draft v0.1 previously circulated)
1.0	28/01/2020	7.2	Details added on derivation of the standardized effect size and NNT statistics
	28/01/2020	7.3	Added clarity on baseline covariate adjustment
	28/01/2020	7.6	Additional exploratory mediation analysis
	28/01/2020	7.8	Addition to sensitivity analysis around baseline imbalance
	28/01/2020	Tables 5-8	Changes to labels / added clarity provided
	28/01/2020	Table 5b	Added

Contents page

Page No.

1. Introduction 8
    1.1 Background and rational 9
    1.2 Aims & Objectives 9
2. Design 11
    2.1 Trial randomisation 11
    2.2 Data collection 11
3. Study & Evaluative Population 15

3.1 Eligibility criteria 15
3.2 Participant identification 15
3.3 Participant flow 15
3.4 Sample for evaluation 16
4. Outcomes 16
4.1 Primary outcome 16
4.2 Secondary outcomes 16
5. Sample size 18
6. Statistical reporting 20
6.1 Participant flow 20
6.2 Attrition 20
7. Methods of analysis 21
7.1 Descriptive statistics 21
7.2 Main analysis of primary outcome 22
7.3 Analysis of secondary outcomes 23
7.4 Sensitivity analysis 23
7.5 Exploratory subgroup analysis of primary outcome 23
7.6 Exploratory mediation analyses 24
7.7 Evaluation of process outcomes 24
7.8 Examination of bias 24

8. Health Economics 26
8.1 Costs 26
8.2 Outcomes 26
8.3 Data analysis 26
9. References 28
10. Figures and Tables 32-51

Glossary of terms / Abbreviations

ADL: Activities of Daily Living
AIC: Akaike Information Criteria
BIC: Bayesian Information Criteria
BNF: British National Formulae
CACE: Complier Average Causal Effect
CEAC: Cost Effectiveness Acceptability Curve
CI: Confidence Interval
CONSORT: Consolidated Standards Of Reporting Trials
CTU: Clinical Trials Unit
DAP: Data Analysis Plan
DMC: Data Monitoring Committee
DoH: Department of Health
EQ-5D: EuroQol 5-dimension Quality of Life measure
GP: General Practice / Practitioner
HSCR: Health & Social Care Research
ICC: Intra-cluster Correlation Coefficient
IQR: Inter-Quartile Range
IT: Information Technology
ITT: Intention-To-Treat
KOOPS-PS: Knee injury and Osteoarthritis Outcome Physical function Short-form
MI: Multiple Imputation
MLM: Multi-Level Model
MSK: MusculoSKeletal
MSK-HQ: MusculoSKeletal Health Questionnaire
NDI: Neck Disability Index
NHS: National Health Service
NICE: National Institute of Clinical Excellence
NRS: Numerical Rating Scale
ONS: Office of National Statistics
PSS: Personal Social Services
P-value: Probability value
QALY: Quality Adjusted Life Year
RCT: Randomised Controlled Trial
RMDQ: Roland-Morris Disability Questionnaire
SAP: Statistical Analysis Plan
SD: Standard Deviation
SMS: Standard Messaging Service
SOP: Standard Operating Procedure
SPADI: Shoulder PAin and Disability Index
STarT MSK: Stratified Tool for MusculoSKeletal disorders
TSC: Trial Steering Committee
TMG: Trial Management Group
TSK: Tampa Scale of Kinesiophobia

Summary Table

**Trial title**	Stratified Primary Care for Musculoskeletal Pain
**Trial design**	A pragmatic, two parallel arm, cluster RCT
**Trial participants**	People with musculoskeletal (MSK) disorders consulting in primary care
**Sample size**	1200
**GP-Practices**	24
**Follow-up**	6 months (with monthly data collection of key outcomes and 6 month questionnaire)
**Primary analysis**	A comparison of average pain intensity across 1-6 months follow-up by longitudinal mixed model regression with adjustment
**Secondary analyses**	A comparison of pain intensity scores at all monthly follow up time-points and secondary health outcomes over/at 6 months follow-up including health economic evaluation (qualitative research is being carried out but is not included in this DAP)

1. Introduction

This document outlines the detailed DATA ANALYSIS PLAN (DAP) for the STarT MSK main trial constituting work-package 4 of the NIHR Stratified Primary Care for Musculoskeletal Pain Programme Grant (NIHR Programme Grants for Applied Research RP-PG-1211-20010). The DAP is intended to be read in conjunction with the current trial protocol (v1.2) – and includes the individual Statistical Analysis Plan (SAP) and Health Economics analyses plans. This DAP is written in conjunction with Keele Health and Social Care Research Standard Operating Procedures (Keele HSCR- SOPs) located at:-

https://www.keele.ac.uk/research/raise/governanceintegrityandethics/healthandsocialcareresearch/

– with particular reference to HSCR- SOP16 (Analysis) and its affiliated documents of TEM33 (DAP Template), GUI13 (Decision Rules for Scoring), POL02 (Data Security Policy) and WI12 (Working Instructions on Data Management Rules), and implemented through the BioStatistics Group affiliated to Keele Clinical Trials Unit (Registered CTU number 36). All aspects of the trial are conducted in accordance with Good Clinical Practice in Clinical Trials (ICH Harmonised Tripartite Guideline 1996) and data management comply with the EU General Data Protection Regulation (GDPR, May 2018).

This DAP will be used as a work description and will guide the Trial Analysts (Statisticians and Health Economists) during the analysis of all quantitative outcomes in order to answer the objectives of the study.

The following committees are established to support trial monitoring and oversight of methodology:-

- Trial Steering Committee (TSC) - consisting of an independent chair and four other independent members. The committee will meet approximately every 6 months from the start of the trial.
- Data Monitoring Committee (DMC) - consisting of an independent chair and two other independent members. The committee will meet approximately every 6 months from the start of the trial.

- Trial Management Group (TMG) – oversee the day-to-day management of the trial and meet on a monthly basis. The TMG include the core members of the team (Chief Investigator, Trial Manager and direct research staff).

1.1 Background and rationale

Musculoskeletal pain such as back, knee, neck, shoulder, or multisite pain is very common. It is the number one reason why people take time off work and accounts for a fifth of all GP consultations. Many people soon recover, but approximately a third of patients still report disabling pain one year after consulting their GP. Early identification of this group of patients as well as finding more effective treatments for them is a high priority.
Stratified care involves clinicians using a short questionnaire to help identify patients who are at risk of having persistent disabling pain and providing recommended matched treatment options. The STarT MSK trial aims to establish if stratified care is more clinically and cost effective compared to usual primary care for patients with these common musculoskeletal pain conditions.

1.2 Aims and objectives

The overall aim of this cluster RCT is to investigate if stratified care, involving the use of a prognostic tool and matched clinical management options, for adults who consult in general practice with one of the five most common MSK pain presentations, is more clinically and cost effective compared to usual non-stratified care.

The specific objectives include both primary objective and secondary objectives and are detailed as follows:

Primary objective:
To determine, in patients presenting with one of the five most common MSK pain presentations in UK primary care, whether stratified care involving use of the Keele STarT MSK Tool to allocate individuals into low, medium and high risk subgroups, and matching these subgroups to recommended matched clinical management options, results in lower pain intensity over 6 months follow-up.

Secondary objectives:
1. Examining differences in secondary outcomes, clinician behaviours and health economic outcomes at 6-months follow-up.

- Patient outcomes include physical function, confidence to manage their pain (pain self-efficacy), psychological distress, levels of fear avoidance beliefs, patient perceived reassurance from their clinician, pain interference with sleep, hobbies/leisure activities, pain interference with work and daily routine, health related quality of life and patient satisfaction with care received (full details of these measurements are provided in trial protocol).
- Clinician behaviours of interest include identifying whether stratified care changes the primary care management of MSK patients. Using the practices’ medical record

data we will examine differences between arms in requests for i) prescriptions (e.g. categorised into such as simple analgesics, non-steroidal anti-inflammatories (NSAIDs), neuromodulators, muscle relaxants, corticosteroid injections and opioids)
ii) referrals (e.g. categorised into physiotherapy/MSK interface services, specialist services including orthopaedics, pain clinics, and rheumatology)
iii) imaging (e.g. categorised into x-rays/MRI scans, MSK ultrasound scans and bone density scans)
iv) sick certifications (e.g. categorised into number per patient and mean length in days)
v) repeat MSK general practice visits
vi) patient self-reported provision of written education/advice from the consultation.
We anticipate that primary care clinical management will involve less variation in clinical behaviours within each risk group and be more in line with stratified care, where patients at low risk of persistent disabling pain are less likely to be referred for additional healthcare whereas patients at medium and high risk are more likely to be referred for additional healthcare in ways that match the recommended management options.

- Health economic evaluation will determine the cost-utility of stratified care in comparison to usual non-stratified care. A cost-consequence analysis will initially be reported, with a subsequent cost-utility analysis from a healthcare perspective to determine cost per quality-adjusted life years (QALY) gained, calculated using EQ-5D-5L responses from the initial and 6 month questionnaires. A broader costing perspective will be considered in a sensitivity analysis, taking into account NHS/Personal Social Services costs and productivity costs associated with time off work. The outcome of interest for the economic analysis will be QALYs. Additional exploratory analyses will consider the cost-effectiveness of stratified care compared to usual non-stratified care for patients at low, medium and high risk of persistent disabling pain.

2. Undertaking a process evaluation in order to explore how stratified care, as a complex intervention, impacts upon service provision, professional practice, and professional-patient interaction. The evaluation will use mixed quantitative and qualitative methods, integrating data both at the collection and analysis stages, in order to generate more detailed and comprehensive findings (details of the analysis of the qualitative data are not provided in this DAP).

2. Design

The STarT MSK trial is a pragmatic, two parallel arm, cluster RCT, with a linked health economic analysis and mixed methods process evaluation in UK primary care (see trial protocol for details).

2.1 Trial randomisation

GP practices are randomised in a ratio of 1:1 to intervention or control using stratified block randomisation based on GP list size using a Keele CTU computer-generated random sequence and concealment by ensuring each practice has an anonymised code. The block

randomisation follows in accordance with the Keele randomisation HSCR- SOP32 – and CHL16 sub-document for cluster trials - and the data sequence is held on a secure server. Access to the allocation sequence is restricted to those with authorisation. The randomisation sequence/stratification is carried out by the senior statistician. Allocation will be shared with the study team (except for the trial statistician and data entry staff who are to remain blind) who will then arrange to inform each practice about their allocation. Data cleaning/checking through stage 1 'data-freeze' and stage 2 'data-lock' reviews (following Keele HSCR-SOP 43 procedures) will be carried out by the trial statistician – thus ensuring that data updating is carried out blind to allocation to retain data integrity.

2.2 Data collection

TAPS is a prospective longitudinal study with three different types of data collection:

a) Individual patient data collected in the following ways:
   i) Clinician-completed computer template at the point of consultation
   ii) Initial and 6 month postal questionnaires to patients (full and minimum data versions)
   iii) Monthly SMS text or one page postal questionnaire to patients
b) Linked medical record data from participating practices
c) Anonymised patient level medical record data from participating practices

a) Individual patient outcomes

i) GP template

The first item on the GP template will be used to identify the location of the MSK pain and how this is answered will determine which letter and questionnaire patients receive in the study, as these will be slightly different for patients with back, neck, knee, shoulder, or those with multi-site pain. The item will ask the GP:

Please confirm the primary pain site the patient is consulting with today:

Possible options for the GPs will include: 'back pain', 'neck pain', 'shoulder pain', 'knee pain', 'multi-site pain', or 'unable to complete template' (which leads to the exit screen).

The second question on the GP template will ask about the intensity of the MSK pain by asking:

How intense was your pain, on average, over the last 2 weeks?

[Responses on a 0-10 scale, where 0 is "no pain" and 10 is "worst pain ever"]

ii) Initial and 6 month follow-up questionnaires

The initial and 6 month follow-up postal questionnaires are designed to collect information on descriptive characteristics of the participants, pain-related characteristics and primary and secondary outcome measures. Further details of the items to be included in each of the postal questionnaires (initial and six months) were summarised in the trial protocol.

Patients will be informed in their invitation letter from the GP practice that this research is to examine how patients with common aches and pain experience their problem, and how this may change over time. They will be told that they have been contacted because they recently visited their GP practice (the date of their visit will be given) for their MSK pain, which will be pre-populated in the letter (e.g. knee pain, shoulder pain etc.) using information from weekly downloaded template codes.

Patients will also be told that it is important they think about their <*MSK pain*> as they answer the following questions.

The first question in the questionnaire will ask:
Thinking about your (e.g. neck) pain: Over the last 2 weeks, on average, how intense was your pain?
[Responses from 0 = no pain to 10 = worst pain ever]

iii) Monthly SMS text or one page questionnaire for 3 items including the primary outcome

Soon after the initial completed questionnaire is received at Keele CTU with written informed consent to follow-up data collection, patients will receive a welcome message to the study via the short message system (SMS) if their preference was to be contacted via text, or via a letter if not, confirming the location of their MSK pain and thanking them for taking part. The monthly one page questionnaire mailings will be sent in an envelope and include a pre-paid reply envelope to ensure patient confidentiality at all times using the patient's initial questionnaire mailing date as the starting point for data collection. If a patient's initial questionnaire response is delayed such that the first monthly communication date is missed, the next consecutive calendar month will be used. However, patients who complete their initial questionnaire (date of patient completion is entered on the questionnaire) more than 4 weeks after their initial mailing date will not be eligible.

In addition to the primary outcome (pain intensity) the monthly SMS text or one page questionnaire includes the following single items for psychological distress and self-efficacy which are taken and adapted with permission from the validated Musculoskeletal Health Questionnaire (Hill et al., 2016):

How much distress have you been experiencing because of your pain, on average, over the last 2 weeks? [0 = no distress to 10 = extreme distress]
How confident have you felt about managing your pain by yourself e.g. medication, changing lifestyle? [0 = not at all confident and 10 = extremely confident]
Trial data will be extracted from source documents and entered onto Keele CTU's in-house data management system. The study and data management teams in Keele CTU will validate and query data for inconsistencies during the course of the trial (as stipulated in Keele HSCR-SOPs 42-44). The trial statistician will verify the data entry against set standards as pre-specified in up-to-date rules relating to WI12 document within SOP43, and conduct any additional validation checks where appropriate at the level of 'data-freeze' and at final data-checking relating to lock-down data ahead of final sign off of data in preparation for analysis (as guided by the aforementioned SOPs). Details of data collected at each time-point are given in Table 1 of the trial protocol (version 1.2).

b) Linked medical records

Clinician clinical decision-making / behaviours will be examined via a review of the practice computerised medical records for all patients who give consent for this (at the end of the initial questionnaire). This will allow data to be analysed from a) individual patient outcomes b) the initial patient-clinician consultation electronic template, c) further aspects of their medical record over 6 months following the MSK consultation. Variables of interest from the MSK consultation will include: the date of consultation, coded reason for the consultation, MSK pain intensity and location, STarT MSK Tool (clinician completed version) individual items

and total score (intervention arm only), and information about the treatments and other actions taken by the clinician. Other clinical behaviours of interest are described in the outcomes section. The information collected on the risk subgroup and management options for patients who have a completed template in the intervention practices will be audited and fed back to clinicians at regular intervals, allowing them to see how closely they have followed the matched clinical management options. At the end of the trial we will also report the fidelity of intervention practices in completing the tool and per protocol treatment for the recommendation matched treatments options (pre-defined). The template MSK pain intensity score will also provide the initial score for the primary outcome for patients in both arms of the trial.

Physiotherapists treating patients referred from participating GP practices will complete their usual clinical records. At the end of the trial the trial team will collect details about the physiotherapy treatment provided for consenting trial participants during the trial period to compare between intervention and control physiotherapy the mean waiting times, mean number of appointments per patient and mean treating therapist grade. These variables will be collected in slightly different ways for each arm. For intervention participants it will be collected individually using a standardised anonymised case report form (with data entered by the treating physiotherapists). For control participants we will ask the physiotherapy service managers to provide means for these three variables for all MSK patients seen in their service (using routinely collected data available in their medical record systems) for the period of time when the trial was active in their service. This activity will be supported by CRN physiotherapy research facilitators where possible.

c) Anonymised patient level medical record data

Each participating GP practice will provide anonymised medical record data from potentially eligible participants who activated the template upon entry of an MSK Read code. We will compare:

- the characteristics of those patients in which the template is activated with those who respond to the initial questionnaire and provide individual level patient outcomes. The information examined will not involve any patient identifiable data and will not be linked to any other data unless prior patient consent has been.
- aspects of clinical behaviours for 6 months following the index consultation to compare intervention and control practices for key treatment process outcomes for each risk subgroup. For example, this will include requests for

  i) prescriptions (e.g. categorised into such as simple analgesics, non-steroidal anti-inflammatories (NSAIDs), neuromodulators, muscle relaxants, corticosteroid injections and opioids)

  ii) referrals (e.g. categorised into physiotherapy/MSK interface services, specialist services including orthopaedics, pain clinics, and rheumatology)

  iii) imaging (e.g. categorised into x-rays/MRI scans, MSK ultrasound scans and bone density scans)

  iv) sick certifications (e.g. categorised into number per patient and mean length in days)

  v) repeat MSK general practice visits

vi) patient self-reported provision of written education/advice from the consultation.

The collection of anonymised and aggregated medical record data is not uncommon within similar general practice research studies that examine potential recruitment bias (Boardman et al., 2005) or for intervention studies examining clinician decision-making and behaviour during the consultation (e.g. POST cluster trial (Mallen et al., 2018) and SWAP cluster trial (Bishop et al., 2014; Wynne-Jones et al., 2018).

3. Study and evaluative population

3.1 Eligibility criteria

The study population consists of adults (aged 18 years and older) who consult a participating GP practice with pain in one of the five most common MSK pain presentations (back, neck, knee, shoulder or multi-site pain).The eligibility criteria are designed to select a group of patients with one of the five most common MSK pain, who do not have indications of serious 'red flag' pathology (e.g. recent trauma with significant injury; acute, red, hot swollen joint; suspected fracture, joint infection or cancer; inflammatory arthropathy such as rheumatoid arthritis; spondyloarthropathy, polymyalgia rheumatic; crystal disease (gout)) and who do not require urgent medical care needs (e.g. Cauda Equina Syndrome). The detailed inclusion and exclusion criteria are listed in the trial protocol.

3.2 Participant identification

The intention of this trial is to recruit 1200 participants (600 per arm). Participants will be identified from the participating general practices. This will be determined from a designated set of Read codes as defined by Jordan et al. (2010) and informed by our pilot RCT. Participating GPs will be trained in the following inclusion and exclusion criteria and given laminated copies of this criteria for their consulting room. The units of randomisation are the general practices and the units of observation are adults consulting for MSK pain with one of the five most common MSK pain presentations.

3.3 Participant flow

A flowchart is designed for updating and data monitoring as the study progresses, showing numbers of the following:

- Numbers of patients approached/screened;
- Numbers of patients invited;
- Number of patients who participated (completed baseline questionnaire and consented for data collection);
- Participants assigned to the different treatments (based on STarT MSK Tool risks in intervention group);
- Participants receiving the intended treatment;
- Loss and exclusion of participants after randomisation (with reasons);
- Participants analysed for each clinical outcome elevation objectives;
- A breakdown for each treatment group.

3.4 Sample for evaluation

Participants who withdraw or who have deviations from the protocol (protocol deviators) will be noted and the precise details will be listed across recruitment/follow-up timelines. Frequency of missing data (and conversely, completion rates) will be reported for each variable at all stages (baseline, monthly feedback (SMS text or 1 page postal questionnaire) and 6-month) by intervention arms. Patterns of missing data will be explored in terms of: (a) timeline of occurrence e.g. does the frequency of missingness increase over time; (b) whether missing data are associated with baseline (socio-demographic and clinical) factors and follow-up pain intensity; (c) if there is differential dropout by trial arm. This evaluation is intended to help provide guidance relating to: (i) appropriateness and frequency of questioning; (ii) possible sensitivity analyses of methods of statistical evaluation and ITT designation. A large imbalance in follow-up rate between trial arms would potentially be problematic and give rise to concerns over possible attrition bias between arms, hence the need to assess overall follow-up rate particularly in relation to monthly pain intensity score returns by trial arm.

4. Outcomes

4.1 Primary outcome

The primary clinical outcome for the trial is the patient reported clinical outcome of pain intensity, measured monthly over 6 months.
"Thinking about your [pain site] pain … Over the last 2 weeks, on average, how intense was your pain?" assessed on a 0-10 Numerical Rating Scale (NRS), where 0=No pain, 10=Pain as bad as could be.

4.2 Secondary outcomes

Secondary clinical outcomes captured at baseline and through monthly (1 to 6 months) follow up (also as potential mediating variables of pain outcome) include:

- Single-item 'distress' measure: "Over the last 2 weeks, on average, how much distress have you been experiencing because of your pain?"; 0-10 NRS, where 0=No distress, 10=Extreme distress
- Single-item 'confidence-in-managing' measure: How confident have you felt about managing your pain by yourself (e.g. medication, changing lifestyle?"; 0-10 NRS, where 0=Not at all confident, 10=Extremely confident)

Secondary clinical outcomes captured at baseline (unless otherwise specified) and/or at 6 months follow up include:

- NRS-Pain (over the last 2 weeks) captured through full 6-month postal response questionnaire.
- Body site specific physical functional measures:

- ➢ The Roland-Morris Disability Questionnaire (RMDQ) for patients with back pain; 0-24 NRS, where 0=No disability, 24=most disability (Roland and Morris, 1983)
- ➢ The Neck Disability Index (NDI) (BenDebba et al., 2002; MacDermid et al., 2009) for patients with neck pain; 0-50 NRS, where 0-4=None; 5-14=Mild; 15-24=Moderate; 25-34=Severe; 35+=Complete (Venron and Mior, 1991)
- ➢ The Shoulder pain and disability index (SPADI) (Breckenridge et al., 2011) for patients with shoulder pain; SPADI total as well as SPADI-Pain and SPADI-Disability sub-scales with scale range 0-100 (0=best score, 100=worst score)
- ➢ The Knee Injury and Osteoarthritis Outcome Score Physical Function Short-form (KOOS-PS) (Perruccio et al., 2008) for patients with knee pain; gives subscale scores for Pain, Symptoms, ADL, Sport/Rec, QOL with scale range of 0-100 (0=worst score, 100=best score)
- ➢ The Short Form 12v2 Physical Component Scale (Ware et al., 2003) for patients with multi-site pain; gives 0-100 scale (0=worst physical health, 100=best physical health)

- Patients’ risk of persistent disabling pain using the Keele STarT MSK Tool (2 versions used: clinical and self-report versions); categories of ‘low’, ‘medium’ and ‘high’ risk
- The MSK pain symptom severity and impact using the Musculoskeletal Health Questionnaire (Hill et al., 2016) (which includes measures of pain interference with sleep, physical activity level, hobbies/leisure activities, work and daily routine); scored from 0-56 (0=worst health, 56=best health)
- Fear of movement using the shortened 11-item version of the Tampa Scale of Kinesiophobia (Archer et al., 2012); 11-item NRS scored from 11-44 (17=no fear-avoidance, 64=complete fear-avoidance)
- [Baseline only] Patient perceived level of reassurance from their clinician will be captured using the Holt and Pincus (2016) reassurance tool, which has four sub-scales: information gathering, relationship building, generic reassurance and cognitive reassurance; subscale scoring 0-21 (0=not at all, 21=a great deal).
- Health related quality of life using the EQ-5D-5L will be used to calculate quality adjusted life years (QALYs) used in the health economic evaluation (Herdman et al., 2011); scale score -0.59 to 1.00 (-0.59=worst health, 1.00 best health) using the NICE-recommended cross-walk tariff.
- [6 months only] Single item question to capture patient satisfaction with care received; 1-5 ordinal scale (1=very satisfied, 2=quite satisfied, 3=no opinion, 4=not very satisfied, 5=not at all satisfied)
- Single-item question asking about days in last week doing moderate physical activities (0 days to 7 days)
- [6 months only] Overall rating of change in their MSK pain since their index consultation (Kamper et al., 2009); scale range -5 to +5 where -5=very much worse, 0=unchanged and 5=completely recovered

- Single-item work performance; NRS 0-10 where 0=Not at all affected, 10=So bad I am unable to do my job
- Time off work in last 6 months; yes/no – and if yes (number of days/ weeks/ months)
- [6 months only] Bespoke resource use questionnaire (for health economic analysis), including visits to health care professionals (primary/ secondary care), treatments/investigations, overnight/inpatient stays due to the MSK problem

For the above multi-item scales the scoring algorithms will follow the criteria specified by the authors of the respective scales as closely as possible. Whereby rules for scoring do not explicitly address incomplete responses/ missing item data, the applied rules for scale scoring will follow the outlined template HSCR-SOP16 GUI13 (section 'Scoring of scales with missing data') (i.e. scale scores may still be calculated if only a small proportion of the items are missing through extrapolation of completed responses).

5. Sample size

The sample size for this cluster RCT has been developed as follows: in an average sized GP practice (6,000 registered adults) about 800 potentially eligible patients will consult with the musculoskeletal pain sites of interest per year, or 400 over 6 months. The feasibility and pilot trial in work package 3 showed that on average the template was activated 375 times over 6 months in each practice, and GPs fully completed it in 41% of cases (154 times or 6 times per week), leading to a letter inviting the patient to participate in the data collection. From the pilot we expect 40% of patients invited will return their initial questionnaire, be eligible and consent to further data collection (or 62 over 6 months in one practice). However, taking a slightly more cautious view given general uncertainty in data and in generalisability of pilot estimates, for purposes of the main trial recruitment we have conservatively estimated the average number of participants recruited per practice within 6 months to be around 33% of those invited (or n=50 in 6 months or n=9 per month per practice).

The trial is powered at 90% to test the hypothesis of overall superiority of stratified care versus usual care based on an alpha of 5% (two-tailed) to detect a small 'effect size' (standardised mean difference) of 0.2 (Cohen, 1988) in the primary outcome (pain intensity) over 6 follow-up time-points (monthly between 1-6 months). An effect size of 0.2 was considered to be appropriate based on information from the feasibility and pilot trial, in which the proportion of responders in the three risk subgroups as *determined from the initial questionnaire* responses was: 32% low risk, 55% medium risk, and 13% high risk. Our previous trial of stratified care compared to control for patients with low back pain found an effect size of 0.3 and 0.4 in the primary outcome (back pain-related physical function) in patients at medium and high risk respectively and so we have assumed these standardised differences in this new trial (Hill et al., 2011). Also, the minimal clinically important difference for the NRS-pain scale in MSK pain has been reported to be 1-point (Busse et al.,2015), which equates to an effect size of about 0.4 relative to an expected SD of about 2.5 (Cohen, 1988). We expect that there would be little or no difference between stratified care compared to control for

patients in the low risk subgroup. Hence, through multiplying these effects by the expected proportion within each of the subgroups: the overall effect size of interest, in context of a superiority trial based on the total study population, is 0.2 (equating to an absolute mean difference of about 0.5 in pain intensity on a 0 – 10 scale).

The sample size calculation takes account of clustering of individual participants by GP practice and likely participant dropout (inflationary effects on sample size requirement) as well as repeated measurements and adjustment for corresponding baseline pain intensity score (deflationary effects). We have allowed for an ICC of 0.01 based on previous patient-level data from primary care trials (Adams et al., 2004) as well as expected variation in recruitment per practice using a guideline coefficient of variation of 0.65 (Eldridge et al., 2006), and together with an expected loss to follow-up across all time-points of approximately 25%, these factors combine to give a sample size inflation factor of ×2.3 (based on an average cluster size of about 50 participants per practice in 6 months). Correlation of data within 6 repeated measurements and correlation of follow-up scores with baseline score are typically 0.7 and 0.5, respectively (Vickers, 2003), which combine to give a sample size deflation factor of ×0.5). The product of inflation and deflation effects result in a magnification of 1.15 compared to a conventional, individual-patient, single follow-up comparison, whereby the sample size requirement would be 525 per treatment arm (or, 1050 in total). The adjusted sample size target is therefore 600 patients per arm (1200 in total) from approx. 24 general practices (approx. 12 per arm). Interim checks of the data will be carried out during the recruitment phase to cross-check assumptions regarding the unknown parameters to monitor the on-going power; additional GP practices will be recruited if necessary to ensure the primary test hypothesis is sufficiently powered.

6 Statistical reporting

Data will be reported according to the reporting guidelines for randomised clinical trials: Consolidated Standards Of Reporting Trials (CONSORT 2010) statement (Schulz et al., 2010; Moher et al., 2010) including extensions to cluster randomised trials (Campbell et al., 2012) and pragmatic trials (Zwarenstein et al., 2008).

Summaries of continuous variables will comprise the number of observations used, mean, median, SD, inter-quartile range, minimum and maximum as appropriate for the distributional form of the data.

Summaries of categorical variables will comprise the number of observations used, and the number and percentage of observations in each category. Tables containing the results of the statistical modelling will present the overall difference between treatment groups with two-sided 95% confidence intervals (CI) and p-values. Hypothesis tests will use a two sided 5% significance level.

6.1 Participant flow

A CONSORT style flow diagram will be used to show the flow of participants through the trial (Figure 1). Included within this are summaries of the reasons patients for declining to take part in the study data collection and reasons for withdrawing from data collection at both levels of GP practice clusters and individual-patient level will be presented (as available).

6.2 Attrition

There are several reasons that a participant may not complete outcome data collection. These include withdrawal of consent, loss to follow-up and death. The number and proportion in each category will be presented by intervention arm. Unless the patient objects, any data collected up to the point of withdrawal would be retained and used in the study analysis. The number of each type of withdrawal will be presented as part of the CONSORT flow diagram and will be summarised in more detail in a separate table which will include the timing and type of withdrawal (**Error! Reference source not found.**).

7. Methods of analysis

The statistical analysis will focus on the following aspects:

7.1). Descriptive statistics

7.2). Main analysis of primary outcome

7.3). Main analysis of secondary outcomes

7.4). Sensitivity analysis

7.5). Subgroup exploratory analysis of primary outcome

7.6). Exploratory mediation analyses

7.7). Evaluation of process outcomes (using medical record data)

7.8). Examination of bias

7.1). Descriptive statistics

7.1.1). Baseline characteristics.

The baseline demographics and clinical characteristics of general practice-clusters and individual participants will be reported. For the continuous variables (e.g. age) either mean and SD will be presented or median and inter quartile range (IQR) depending on the distribution of the data. The number of observations used in each calculation will be presented alongside the summaries. For the categorical variables (e.g. sex), the number and percentage of participants in each of the categories and the total number of observations will be presented. The questionnaires include items for the two versions of the Keele STarT MSK Tool (clinical and self-report versions); the descriptive statistics around number and percentage in 'low', 'medium' and 'high' risk subgroups across both versions will be provided (as both are considered relevant for different aspects). A comparison of the responses, overall scoring and sub-group numbers will be undertaken.

All baseline summaries will be presented and reported for each intervention arm and in total (**Error! Reference source not found.** and 2). CONSORT guidelines generally do not recommend statistical significance testing of baseline imbalances between the intervention arms; however, in line with a recent publication which suggests doing so for cluster-RCTs we will carry out baseline testing of individual level characteristics to examine the level of selection bias as indicated by potential imbalances in baseline covariates between intervention arms (Bolzern et al, 2019).

7.1.2). Follow-up summary scores

Descriptive summary data will be presented in the form of mean or median values along with standard deviation or interquartile range values for numerical data, and frequency counts (percentages) for categorical data. Numbers of responses will be reported across summary scales – in total and by intervention arm (see Table 8).

7.2). Main analysis of primary outcome

To avoid any potential bias in the analysis, Intention to treat (ITT) will be the primary analysis population (including primary, secondary and safety outcomes) unless otherwise stated. This is defined as general practice-clusters (and affiliated participants) being analysed as they are randomised regardless of the intervention. Data for individuals who withdraw consent will be included up to the point of withdrawal. Primary analysis will compare mean difference in pain intensity scores between trial arms over six months follow-up using a hierarchical linear mixed regression model evaluating repeated measures data at 1, 2, 3, 4, 5 and 6 months follow-up (level-1) within individuals (level-2) and taking into account clustering of individuals within general practices – the unit of randomisation (level-3). The analyses will be adjusted for age, sex and baseline pain intensity score (recorded from the IT template at the point of consultation) at the individual-patient level, and general practice size. This analysis fulfils the ITT principle with analysis as randomised and missing data being accounted for under the missing at random assumption. Although the primary analysis will focus on the 'average' intervention effect across 1-6 months follow-up, treatment by time interaction terms evaluate between-arm differences in mean responses across each of the individual time-points of 1, 2, 3, 4, 5 and 6 months. STATA command "mixed" will be used to perform this hierarchical linear mixed regression model. Model fit will be assessed across difference covariance structures (unstructured, independence, exchangeable, autoregressive) to ascertain the best-fit model that will be implemented – i.e. the model that gives the lowest BIC, AIC and highest log-likelihood statistics (from the STATA *estat ic* command). As well as reporting mean differences (on the unstandardized scale) we will report standardized effect sizes (relative to the overall baseline SD). Further, we will calculate the number (percent) in each arm that improve by at least 1-point on the NRS-pain scale across all timepoints and on average compared to baseline (equivalent margin change to the MCID). The absolute percent differences will then be used to derive the Number-Needed-To-Treat (NNT) statistics through calculation of the reciprocal of the absolute percent difference between groups.

The monthly pain intensity scores are used but if, for any individual, the last monthly SMS/brief questionnaire response is missing but they have completed the corresponding pain intensity question in their returned 6-month questionnaire (if completed within 20 days of the date of issue of their monthly SMS/brief questionnaire) then the available pain intensity score response will be used (as the 6-month score) for purposes of the primary outcome evaluation (see Table 5).

7.3). Analysis of secondary outcomes

Analysis of secondary outcomes will similarly be carried out following the ITT approach and using a linear mixed model for numerical outcomes and generalised mixed logistic models for categorical outcomes e.g. derived through the STATA command "meglm" to perform a multilevel generalised linear model (adjusted for age, sex, baseline pain (GP template) and corresponding baseline score (if applicable) at the individual-patient level, and general practice size). For monthly follow-up measures of distress and confidence in managing pain, the analysis will follow that of the primary analysis with initial focus on 'average' scores over the 6 months of follow-up and then the time-specific between-arm estimates. The focus of the other secondary measures is on 6-month follow-up data only.

7.4). Sensitivity analysis

A sensitivity analysis will be carried out using a complier average causal effect analysis (CACE) to provide an unbiased estimate of intervention effect for patients treated according to the stratified care 'protocol' i.e. for the intervention arm 'protocol' is taken as clinical management selection according to the Keele STarT MSK Tool matched treatment recommendations (per protocol 'flagging' is descriptively shown in Table 3). CACE analysis will be performed using a 2-step instrumental variable regression modelling approach where the first step relates to model prediction of 'compliance' (at level 2 (individual patient level)) using study arm only as a fixed-effect predictor and practice and participant ids as random-effects, and the second step estimates the between-arm difference in outcome ('average' pain intensity) based on predicted compliance – the endogenous (instrumented) variable (from the first step) and the exogenous (instrumental) variables of study arm, age, gender and point-of-consultation pain score using a mixed effects model as used in the primary analysis (Section 7.2) (see footer of Table 5 for reporting format).

7.5). Subgroup exploratory analysis of primary outcome

Subgroup exploratory analysis of the primary outcome ('average' pain intensity) will be carried out by modelling intervention arm-interaction terms within the regression models for: (i) risk-subgroups (low (reference category), medium and high risk); (ii) single MSK pain (reference) site versus multi-site pain; (iii) pain site (back (reference), shoulder, knee, neck). Subgroup analysis will be performed regardless of the results of the primary analysis. The mean between-arm difference (and 95% CI and P-value) will be computed for each subgroup comparison and visually displayed via a forest plot. The main focus will be on the 'average' pain intensity rather than on 3-way interactions of intervention-subgroup-time – but the 3-way interaction results will also be examined (and descriptive results produced by subgroup). We will not calculate separate p-values within each subgroup category (Wang et al. 2007). [See Tables 6a and 6b and Figure 3 for illustration of data reporting format]

7.6). Exploratory mediation analyses

If there is a significant between-group difference in the primary outcome (overall NRS-Pain) then we will carry out exploratory mediation analysis to examine (i) which treatment modalities are 'causal' in effect (treatment modalities are listed in Table 4); (ii) if psychological mediators (month 1-5 NRS- psychological stress pain self-efficacy) are on the causal pathway for effect; (iii) if patient perceived reassurance post GP consultation mediates health outcome. This will be performed using structural equation modelling methods – carried out through (e.g. 'sem'/'gsem' commands in STATA). Exploration will focus on direct/indirect associations of intervention effect on 6-month pain outcome.

7.7). Evaluation of process outcomes

Process outcomes will be evaluated through comparison of aggregated anonymised data at the level of the participating general practices, by examining, for example, re-consultation rates for MSK pain over 6 months and referral rates between practices in the stratified care versus usual care arms, and in the stratified care arm the proportions of patients for whom the electronic template is completed and matched clinical management options are selected overall and stratified by risk subgroup (as shown in Tables 3 and 4).

As available, a descriptive analysis will be undertaken on physiotherapist data by treatment arm. Of interest will be the average date of start of treatment, frequencies for grade of main physiotherapist, and the median (range) as well as frequency counts for the number of treatments provided.

7.8). Examination of bias

Selection bias will be examined through scrutiny of comparability of recruitment rates per trial arm and comparability in practice and participant characteristics (shown in Figure 1 and Tables 1 and 2). Further, a comparison will be performed examining the characteristics of patients in which the electronic template is activated but who did not take part in the study (non-participants) with those who did participate in terms of practice distribution, aggregate pain intensity scores, location of MSK pain, and (within the intervention arm) the proportion of patients at low, medium and high risk (from the GP-consultation template. Both crude descriptive and inferential statistics will be reported.

Differential attrition between-study arms will be examined and reported descriptively: frequencies for responses by study arm will be recorded in the descriptive tables. We will examine pattern of association of follow up response. We will compare baseline socio-demographic and clinical variables and (for response≥1) monthly NRS-Pain follow up scores across level-of-completion of NRS-Pain (level of completion = 0 to 6, where 0=non-response, 1=responded once and 6=responded to all six monthly follow ups) to ascertain whether pattern of missingness is likely to be 'missing completely at random', 'missing at random' or 'not missing at random'. If the overall follow up rate of the primary outcome is over 5% different between study arms and/or there is a clear baseline imbalance between arms (otherwise the impact is likely to be very small) and pattern of missing data is 'missing at random' (beyond the included baseline variables in the main analysis) then we will undertake a multiple imputation (via chained equations) analysis inclusive (in addition) of baseline variables that are observed to be statistically associated with follow up response and/or imbalanced. Further, if the pattern of missingness is seen to suggest that it is non-ignorable the MI sensitivity analysis will address missing data imputations with additionally incremented or reduced value corresponding to the overall baseline SD (thereby mimicking the non-ignorable pattern).

Final analysis will be carried out after all the data are collected, entered and cleaned. STATA (least, version 15) and/or R (least, version 3.6) and/or SPSS (least, version 24) will be used to carry out statistical programming and analyses. Inferential analyses will include reporting of the main (point) estimate for the mean difference (numerical outcomes) or odds ratio (categorical outcomes) along with 95% confidence intervals and P-values (two-tailed). Odds ratios will also be converted to absolute risk differences (using the Usual Care prevalence as the base-reference in any conversion).

Section 6-7 data review and analysis will be performed independently by the two named statisticians using the SAP within this DAP as a guide to programming and analysis from data lockdown through to calculation of results. The two statisticians will work through any results discordance(s) and if consensus agreement cannot be reached between the two statisticians on any of the discordant findings then a third (independent) statistician will be asked to review and resolve any differences.

8. Health Economics

The health economics analysis will determine the cost-effectiveness of stratified care in comparison to usual, non-stratified care over 6 months. A cost-consequence analysis will

initially be reported, describing all the important results relating to costs and consequences. Subsequently cost-utility analysis will be undertaken from an NHS/Personal Social Services (PSS) perspective to determine the cost per additional QALY gained. A broader costing perspective will be considered in a secondary analysis, taking into account NHS/PSS costs, private MSK-related healthcare costs and productivity costs associated with time off work.

8.1 Costs

Resource use information will be obtained on primary care consultations (general practitioners and practice nurses), secondary care consultations (e.g. hospital consultants, physiotherapists), prescriptions, hospital-based procedures (diagnostic tests, injections, and investigations) and length of inpatient stays, and surgery. Patients will be asked to distinguish between UK NHS and private provision. Cost data will be collected via participant questionnaire at six months. Unit costs will be obtained from standard sources and healthcare providers including the British National Formulary (BNF), Unit Costs of Health and Social Care and NHS Reference costs (BNF 2017; DoH 2017 ;Curtis and Burns, 2017).

Given that musculoskeletal conditions are associated with significant loss of productivity costs, information will also be collected from participants on occupation status, time off work related to their musculoskeletal problem and reduced work performance (presenteeism). This will enable the calculation of productivity costs, allowing analysis from a broader societal cost perspective. The average wage for each respondent will be identified using UK Standard Occupational Classification coding and annual earnings data for each job type (ONS, 2017).

8.2 Outcomes

The outcome of interest for the economic analysis is quality-adjusted life years (QALYs) and will be generated from participant responses to the EQ-5D 5L questionnaire at baseline and at six months follow-up. The crosswalk value set will be applied to patient responses to obtain utility scores, in line with current NICE recommendations.

8.3 Data analysis

The cost-utility analysis will be carried out on an intention to treat basis, with the aim of estimating the difference in costs and QALYs between the stratified care and usual, non-stratified care arms. Missing EQ-5D 5L and cost data will be imputed using multiple imputation techniques (Rubin, 1987) in order to ensure that all trial participants are included in the final analysis. For each participant, a QALY score over the 6 month follow up period will be estimated using the area under the curve approach (Mathews et al. 1990). Imbalances in baseline utility (EQ-5D 5L) scores between the stratified care and usual non-stratified care arms will be controlled for using a regression approach (Manca et al. 2005).

Total health care costs over the study period will be calculated by multiplying the resource items used by the respective unit cost and summing over all items. Differences in mean costs and QALYs between the stratified care and usual non-stratified care arms will be estimated. The data for costs is likely to have a skewed distribution; therefore, a non-parametric comparison of means (e.g. bootstrapping) will be undertaken to estimate 95% confidence intervals around costs.

Due to the nature of the trial, methods are required to address clustering in both costs and outcomes, and to recognise correlation between individual- and cluster-level costs and outcomes. Methods currently suggested in the health economics literature are multilevel models (MLM) and the 2-stage non-parametric bootstrap (TSB). (Gomes, 2011). For the base

case scenario, MLM will be used to estimate differential costs, differential QALYs and incremental net benefits. The analysis will also allow us to control for covariates. The robustness of the results will be explored using sensitivity analysis. This will explore uncertainties in the trial data itself as well as the methods employed to analyse the data. A cost-effectiveness acceptability curve (CEAC) will be constructed to assess the probability that stratified care is effective at different willingness-to-pay thresholds. In order to estimate productivity costs, self-reported days off work will be multiplied by the average wage rate. The analysis will use the human capital approach.
Planned sensitivity analysis will include: 1) a complete-case analysis as an alternative to using an imputed dataset 2) a broader societal perspective 3) Additional exploratory analyses that will consider the cost-effectiveness of stratified care versus usual non-stratified care for patients in the low, medium and high risk subgroups separately, a strategy previously used in stratified care for back pain. All analyses will be performed using performed using Stata 15 software.

9. References

Adams, G., et al., Patterns of intra-cluster correlation from primary care research to inform study design and analysis. J Clin Epidemiol, 2004. 57(8): p. 785-94.

Archer, K.R., et al., Comparative study of short forms of the Tampa Scale for Kinesiophobia: fear of movement in a surgical spine population. Arch Phys Med Rehabil, 2012. 93(8): p. 1460-2.

BenDebba, M., et al., Cervical spine outcomes questionnaire: its development and psychometric properties. Spine (Phila Pa 1976), 2002. 27(19): p. 2116-23; discussion 2124.

Bishop A, Ogollah RO, Jowett S, Kigozi J, Tooth S, Protheroe J, Hay EM, Salisbury C, Foster NE; STEMS study team. STEMS pilot trial: a pilot cluster randomised controlled trial to investigate the addition of patient direct access to physiotherapy to usual GP-led primary care for adults with musculoskeletal pain. BMJ Open. 2017 Mar 12;7(3):e012987.

Bishop, A., et al., Rationale, design and methods of the Study of Work and Pain (SWAP): a cluster randomised controlled trial testing the addition of a vocational advice service to best current primary care for patients with musculoskeletal pain (ISRCTN 52269669). BMC Musculoskelet Disord, 2014. 15: p. 232.

Boardman, H.F., et al., A method to determine if consenters to population surveys are representative of the target study population. J Public Health (Oxf), 2005. 27(2): p. 212-4.

Bolzern JE, Mitchell A, Torgerson DJ. Baseline testing in cluster randomised controlled trials: should this be done? BMC Med Res Methodol. 2019;19(1):106.

Breckenridge, J.D. and J.H. McAuley, Shoulder Pain and Disability Index (SPADI). J Physiother, 2011. 57(3): p. 197.

Busse JW, Bartlett SJ, Dougados M, Johnston BC, Guyatt GH, Kirwan JR, Kwoh K, Maxwell LJ, Moore A, Singh JA, Stevens R, Strand V, Suarez-Almazor ME, Tugwell P, Wells GA. Optimal Strategies for Reporting Pain in Clinical Trials and Systematic Reviews: Recommendations from an OMERACT 12 Workshop. J Rheumatol. 2015; 42(10):1962-1970.

Campbell MK, Piaggio G, Elbourne DR, Altman DG, for the CONSORT Group. Consort 2010 statement:extension to cluster randomised trials. BMJ 2012; 345:e5661doi: 10.1136/bmj.e5661.

Cohen, J., Statistical power analysis for the behavioral sciences. 2nd ed, ed. N. Hillsdale. 1988: Lawrence Earlbaum Associates.

Curtis L, Burns A. PSSRU: Unit Costs of Health & Social Care. University of Kent, Canterbury, United Kingdom: Personal Social Service Research Unit (PSSRU); 2017.

Department of Health. National Schedule of Reference Costs: 2016–2017. London: Department of Health; 2017.

Eldridge, S.M., D. Ashby, and S. Kerry, Sample size for cluster randomized trials: effect of coefficient of variation of cluster size and analysis method. Int J Epidemiol, 2006. 35(5): p. 1292-300.

European Commission. General Data Protection Regulation (GDPR). https://ec.europa.eu/commission/sites/beta-political/files/data-protection-factsheet-changes_en.pdf

Gomes M, Ng ES, Grieve R, Nixon R, Carpenter J, Thompson SG. Developing appropriate methods for cost-effectiveness analysis of cluster randomized trials. Med Decis Making 2012; 32:350–61.

Herdman M, Gudex C, Lloyd A, Janssen M, Kind P, Parkin D, Bonsel G, Badia X. Development and preliminary testing of the new five-level version of EQ-5D (EQ-5D-5L). Qual Life Res, 2011. 20(10): p. 1727-36.

Hill, J.K., S. Benedetto, E. Myers, H. Blackburn, S. Smith, S. Dunn, K. Hay, EM. Rees, J. Beard, D. Glyn-Jones, S. Barker, K. Ellis, B. Fitzpatrick, R. Price, A, Development and initial validation of the Arthritis Research UK Musculoskeletal Health Questionnaire (MSK-HQ) for use across musculoskeletal care pathways. BMJ Open, 2016.

Holt N, Pincus T. Developing and testing a measure of consultation-based reassurance for people with low back pain in primary care: a cross-sectional study. BMC Musculoskelet Disord. 2016; 17:277.

International Conference on Harmonisation of Technical Requirements for Registration of Pharmaceuticals for Human Use. ICH Harmonised Tripartite Guideline. Guideline For Good Clinical Practice E6(R1). Version 4. 1996.

Joint Formulary Committee. BNF - British National Formulary. London: BMJ Group and Pharmaceutical press; 2017.

Jordan, K.P., et al., Annual consultation prevalence of regional musculoskeletal problems in primary care: an observational study. BMC Musculoskelet Disord, 2010. 11: p. 144.

Kamper, S.J., C.G. Maher, and G. Mackay, Global rating of change scales: a review of strengths and weaknesses and considerations for design. J Man Manip Ther, 2009. 17(3): p. 163-70.

Keele Health & Social Care Research Standard Operating Procedures (HSCR-SOPs) https://www.keele.ac.uk/research/raise/governanceintegrityandethics/healthandsocialcare research/

Mallen, C. Mallen CD, Nicholl BI, Lewis M, Bartlam B, Green D, Jowett S, Kigozi J, Belcher J, Clarkson K, Lingard Z, Pope C, Chew-Graham CA, Croft P, Hay EM, Peat G. The effects of implementing a point-of-care electronic template to prompt routine anxiety and depression screening in patients consulting for osteoarthritis (the Primary Care Osteoarthritis Trial): A cluster randomised trial in primary care. PLoS Med. 2017 Apr 11;14(4):e1002273.

Manca A, Hawkins N, Sculpher M. Estimating mean QALYs in trial-based cost-effectiveness analysis: The importance of controlling for baseline utility Article in Health Economics. Health Econ 2005; 14:487–96.

Matthews JN, Altman DG, Campbell MJ, Royston P (1990) Analysis of serial measurements in medical research British Medical Journal 300, 230-235

Moher D, Hopewell S, Schulz KF, Montori V, Gøtzsche PC, Devereaux PJ, Elbourne D, Egger M, Altman DG. CONSORT 2010 explanation and elaboration: updated guidelines for reporting parallel group randomised trials. BMJ 2010;340:c869doi: 10.1136/bmj.c869.

Office for National Statistics. Annual survey of hours and earnings. n.d. URL: http://www.ons.gov.uk. 2017. (Accessed 1 March 2019).

Perruccio, A.V., et al., The development of a short measure of physical function for knee OA KOOS-Physical Function Shortform (KOOS-PS) - an OARSI/OMERACT initiative. Osteoarthritis Cartilage, 2008. 16(5): p. 542-50.

Roland, M. and R. Morris, A study of the natural history of back pain. Part I: development of a reliable and sensitive measure of disability in low-back pain. Spine (Phila Pa 1976), 1983. 8(2): p. 141-4.

Schulz,KF, Altman DG, Moher D, for the CONSORT Group. CONSORT 2010 Statement: updated guidelines for reporting parallel group randomised trials. BMJ 2010;340:c332doi: 10.1136/bmj.c332.

Vernon, H. and Mior, S. The Neck Disability Index: A study of reliability and validity. Journal of Manipulative and Physiological Therapeutics, 1991, 14, 409-415

Vickers, A.J., How many repeated measures in repeated measures designs? Statistical issues for comparative trials. BMC Med Res Methodol, 2003. 3: p. 22.

Wang, Rui, Stephen W. Lagakos, James H. Ware, David J. Hunter, and Jeffrey M. Drazen. 2007. "Statistics in Medicine — Reporting of Subgroup Analyses in Clinical Trials." New England Journal of Medicine 357 (21): 2189–94. https://doi.org/10.1056/NEJMsr077003

Ware, J.E., Jr., SF-36 health survey update. Spine (Phila Pa 1976), 2000. 25(24): p. 3130-9.

Wynne-Jones G, Artus M, Bishop A, Lawton SA, Lewis M, Jowett S, Kigozi J, Main C, Sowden G, Wathall S, Burton AK, van der Windt DA, Hay EM, Foster NE; SWAP Study Team. Effectiveness and costs of a vocational advice service to improve work outcomes in patients with musculoskeletal pain in primary care: a cluster randomised trial (SWAP trial ISRCTN 52269669). **Pain** 2018; 159(1):128-138.

Zwarenstein M, Treweek S, Gagnier JJ, Altman DG, Tunis S, Haynes B, Oxman AD, Moher D. Improving the reporting of pragmatic trials: an extension of the CONSORT statement. BMJ 2008; 337;a2390

10. Tables/Figures

Figures:-

Figure 1: Study flowchart
Figure 2: Summary of NRS-Pain by intervention arm (from baseline through 6-month follow up)
Figure 3: Forest plot summary of pre-planned subgroup analyses for NRS-Pain (showing overall and monthly follow up mean differences (95% CI) across pre-defined subgroup analyses)
Figure 4: Cost-effectiveness uncertainty base-case analysis
Figure 5: Sensitivity Analysis: Complete-case analysis

Tables:-

Table 1: Practice characteristics (and related)
Table 2: Individual baseline participant characteristics
Table 3. GP fidelity to the recommended matched management options (Stratified Care arm)
Table 4. Comparison of GP decision-making between intervention and control practices
Table 5a: Summary of overall and monthly follow up NRS-Pain (primary outcome measure) scores and between-group differences
Table 5b: Number (%) achieving the MCID for the NRS-Pain (1-point change) and between-group differences
Table 6: Subgroup analyses of NRS-Pain (6a) Descriptive summary mean (SD) scores by STarT MSK risk subgroups (6b) Descriptive summary mean (SD) scores by pain region
Table 7: Summary of overall and monthly follow up NRS-psychological distress and NRS-pain self-efficacy (secondary outcome measures)
Table 8: Summary of secondary health outcomes from the 6 month follow up questionnaire
Table 9: Unit costs of resources
Table 10: Summary of resource use and costs by study arm
Table 11: Summary of work-related outcomes for participants in paid employment at baseline, by intervention arm
Table 12: Summary of aggregated costs (£) by intervention arm and between-group differences.
Table 13: Descriptive and incremental health outcomes over 6 months for the base-case and complete-case analyses.
Table 14: Subgroup analysis: Costs and health outcomes mean (SD) scores by STarT MSK risk subgroups (Imputed analysis)

Figure 1: Study flowchart

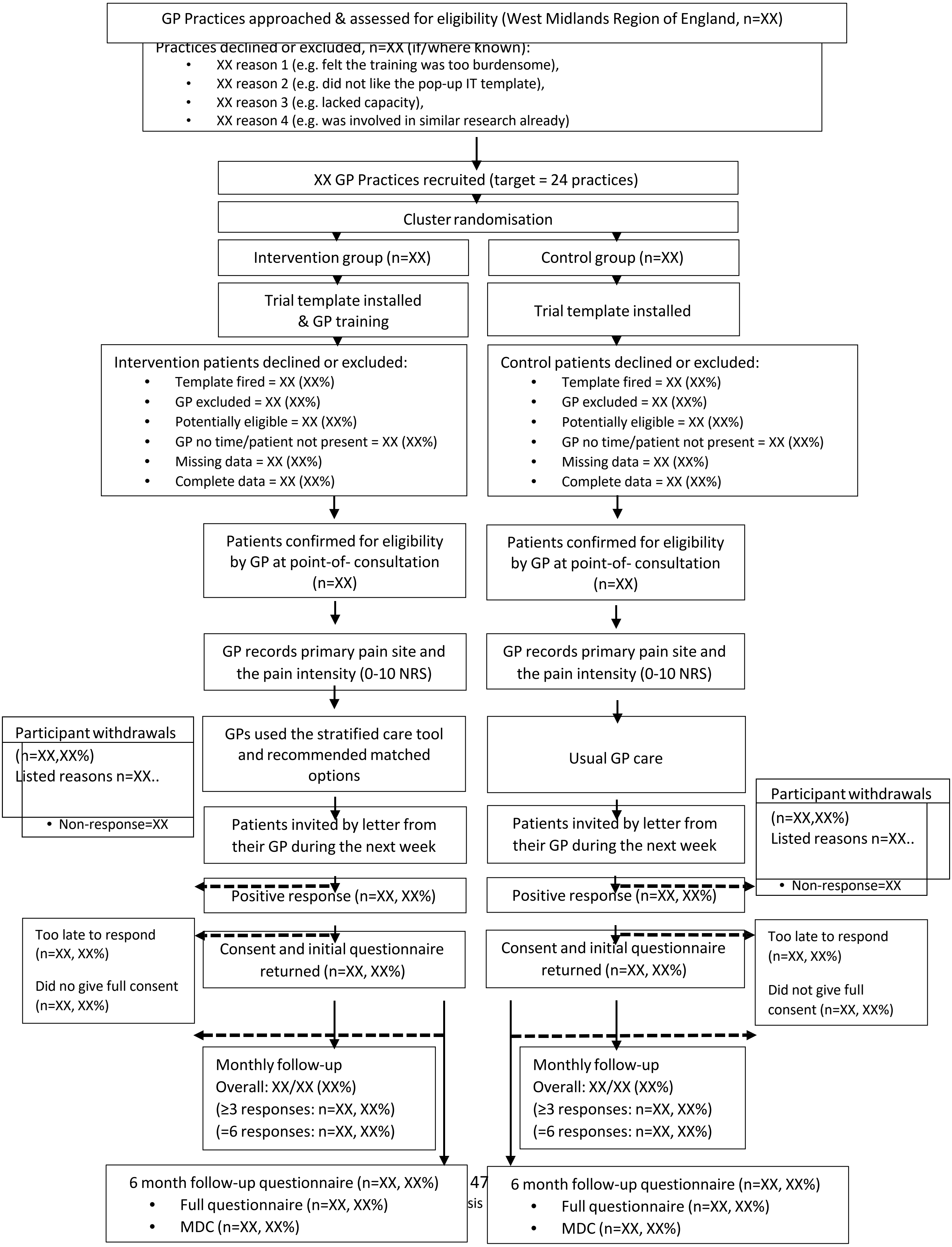

Table 1: Practice characteristics (and related)

Key characteristics	SC (n=XX)	UC (n=XX)
Registered population size,		
- mean (SD)		
- median (IQR; minimum, maximum)		
English Indices of Area Deprivation (2019)*, median (IQR)		
Index of Multiple Deprivation (IMD),		
Income deprivation		
Employment deprivation		
Education, Skills & Training deprivation		
Health deprivation & Disability		
Crime		
Barriers to Housing & Services		
Living Environment deprivation		
Index of Multiple Deprivation [IMD]		
Other possible characteristics#		

* English Indices of Area Deprivation: The 32,844 Lower-layer-Super-Output Areas in England are ranked according to their deprivation score. For each of the neighbourhood-level Indices, the most deprived Lower-layer Super Output Area in England is given a rank of 1, and the least deprived a rank of 32,844 (IMD is an aggregate of the seven sub-indices).
# Any further cluster variables collected related to the baseline Practice-level e.g. average grade of referred physiotherapists.

Table 2: Individual baseline participant characteristics

Key characteristics	n_{SC}, n_{UC}	Total	SC	UC	P-value
Age (years), mean (SD)					
Gender, n (%)					
Male					
Female					
Other					
Point-of-Consultation with GP					
NRS-Pain, mean (SD)					
STarT MSK tool (Intervention arm only), mean (SD)				-	-
STarT MSK (Intervention arm only) - risk subgroup, n (%)				-	-
Low risk (0-4 score)					
Medium risk (5-8 score)					
High risk (9-12 score)					
Baseline questionnaire					
Ethnicity, n (%)					
Mixed					
Asian					
Black					
White					
Other					
Prefer not to say					
Lives alone, n (%)					
No					
Yes					
Currently employed, n (%)					
No					
Yes					
Non-manual, n (% of Yes)					
Manual					
Social class (SOC2000 Major classification), n (%)					
1 Managers & Senior Officials					
2 Professional Occupations					
3 Associate Professional & Technical Occupations					
4 Administrative and Secretarial Occupations					
5 Skilled Trades Occupations					
6 Personal Service Occupations					
7 Sales and Customer Service Occupations					
8 Process, Plant and Machine Operatives					
9 Elementary Occupations					
Performance at work (0-10 NRS)#, mean (SD)					
Time off work, n (%)					
No					
Yes					
No. of days off (of Yes subgroup), median (IQR)					

Health Literacy – need help, n (%)
  Never
  Rarely
  Sometimes
  Often
  Always
Pain Area affected, n (%)
  Knee
  Neck
  Back
  Shoulder
  Multisite Pain
Pain intensity (0-10 NRS)#, mean (SD)
Distress (0-10 NRS)#, mean (SD)
Confidence to manage (0-10 NRS)#, mean (SD)
Pain duration, n (%)
  < 3 months
  3-6 months
  7-12 months
  1-2 years
  3-5 years
  6-10 years
  >10 years
Overall pain change (-5 to +5)#, mean (SD)
No. of previous episodes in last 3 years, n (%)
  0
  1
  2-3
  4-9
  10+
Previous surgery related to problem, n (%)
  0
  1
  2
  3+
Days of moderate activity in last week, median (IQR)
Physical Function, mean (SD)
  Back (RMDQ)
  Neck (NDI)
  Shoulder (SPADI-Function subscale)
  Knee (KOOS-PS)
  Multi-site (SF12 PCS)
  Standardised function scale (0, 1)#
MSK-HQ (0-56)#, mean (SD)
STarT MSK tool (Clinical version), mean (SD)
STarT MSK (Clinical version) - risk subgroup, n (%)
  Low risk (0-4 score)

Medium risk (5-8 score)
High risk (9-12 score)
STarT MSK tool (Self-report version), mean (SD)
STarT MSK (Self-report version) - risk subgroup, n (%)
Low risk (0-4 score)
Medium risk (5-8 score)
High risk (9-12 score)
Health-related Quality of Life (EQ-5D), mean (SD)
Fear Avoidance behaviour (TSK-11), mean (SD)
No. of listed long term conditions, n (%)
0
1
2
≥3
Receipt of written information from GP, n (%)
Perceived reassurance from GP consultation (ECRQ), mean (SD)
Data gathering
Relationship building
Generic
Cognitive
Total
Satisfaction with initial GP care, n (%)
Very satisfied
Quite satisfied
No opinion
Not very satisfied
Not at all satisfied
Preferential mode of follow up, n (%)
Text
Post

SC = Stratified Care; UC = Usual Care. n_{SC} = number of data analysed in the SC arm; n_{UC} = number of data analysed in the UC arm.

Performance at work (0-10 NRS) - where 0=problem ‘not at all’ affected performance over last 6 months, 10=’so bad I am unable to do my job’; NRS-Pain 0=’no pain’, 10=’worst ever pain’; NRS-Distress 0=’no distress’, 10=’extreme distress’; NRS-Confidence to manage 0=’not at all confident’, 10=’extremely confident’; MSK-HQ = Musculoskeletal Health Questionnaire (0-56 scale) based on summation of 14-items on a 0-4 scale and where 0=worst musculoskeletal health status and 56=best musculoskeletal health status; Pain change scale 11-point NRS scale (-5 to +5) where -5=very much worse, 0=unchanged, +5=completely recovered [change from clinic appointment to time of self-report baseline completion]; RMDQ = Roland & Morris Disability Questionnaire (0-24 scale) where 0=no low back pain/disability, 24=maximum low back/pain disability; NDI = Neck Disability Index (0-50 scale) where 0=no disability, 50=maximum disability; SPADI = Shoulder Pain & Disability Index (0-100) 0=no disability, 100=maximum disability); KOOS-PS = Knee Injury and Osteoarthritis Outcome Score Physical Function Short-form (0-100) 0=extreme disability, 100=no disability; SF12-PCS = Short Form 12v2 Physical Component Scale (0-100) 0=worst physical health score, 100=best physical health score. ECRQ …; TSK-11 = Tampa Scale of Kinesiophobia (11-44) 11=minimum fear avoidance, 44=maximum fear avoidance; STarT MSK Tool score (0-12) 0=lowest risk, 12=highest risk.

P-value will be derived through linear or generalised mixed model accounting for GP-Practice clustering (random factor).

[Note: Categories may be pooled for purpose of final presentation e.g. ethnicity (white / non-white= mixed or black or asian or other); social class (1-3 / 4-9); pain duration (<6 weeks / 6-12 weeks / 12+ weeks); satisfaction (very satisfied / quite satisfied / not satisfied=no opinion or not very satisfied or not at all satisfied); health literacy (never or rarely / sometimes or often or always)]

Table 3. GP fidelity to the recommended matched management options (Stratified Care arm)

Values	Low Risk	Medium Risk	High Risk	Grand Total
	(n=XX, XX%)	(n=XX, XX%)	(n=XX, XX%)	
Verbal advice				
Written advice				
OTC Medication				
MSK referral				
Physio referral				
Exercise programme				
Expert peer support				
Lifestyle				
Opioid medication				
Corticosteroid inj.				
Pain Management referral				
Referral to secondary care				
Refer for imaging				
Prescribed atypical analgesia				
Address comorbidities				
	XX	XX	XX	XX

Fidelity to stratified care in decision-making	n (%)
Per protocol (Overall)	
Low risk - per protocol	
Medium risk - per protocol	
High risk - per protocol	
Against protocol	
Low risk - given Medium treatments	
Low risk - given High treatments	
Low risk – only tool used (no treatments selected)	
Medium risk – given Low treatments	
Medium risk - given High treatment	
Med risk – only tool used (no treatments selected)	
High risk – given Low treatments only	
High risk – given Medium treatments	
High risk – only tool used (no treatments selected)	
Grand Total	

Table 4. Comparison of GP decision-making between intervention and control practices

	Usual Care practices	Stratified Care practices				
	All patients (n=XX)	All patients (n=XX)	All partici-pants (n=XX)	Low risk† (n=XX)	Med risk† (n=XX)	High risk† (n=XX)
**During the first week** of MSK pain index GP consultation (0-7 days)						
**Prescription**, n (n per patient)						
Simple analgesics						
Weak and strong opioids						
Anti-inflammatories						
Neuromodulators						
Muscle relaxants						
Corticosteroid injection						
**Referral**, n (n per patient)						
Physiotherapy or MSK interface clinic						
Specialist orthopaedics						
Pain clinic						
Rheumatology						
**Imaging**, n (n per patient)						
MSK X-ray or MRI						
MSK ultrasound scan						
Bone density scan						
**Sick certification**						
Sick certification, n (n per patient)						
Sick cert. length, median days (IQR)						
**Over the next 6 months** after the first week of index MSK pain consultation (8-180 days)						
**Prescription**, n (n per patient)						
Simple analgesics						
Weak and strong opioids						
Anti-inflammatories						
Neuromodulators						
Muscle relaxants						
Corticosteroid injection						
**Referral**, n (n per patient)						
Physiotherapy or MSK interface clinic						
Specialist orthopaedics						
Pain clinic						
Rheumatology						
**Imaging**, n (n per patient)						
MSK X-ray or MRI						
MSK ultrasound scan						
Bone density scan						
**Sick certification**						
Sick certification, n (n per patient)						

	Sick cert length, median days (IQR)						
**Repeat MSK GP consultations over 6 months** (8-182 days), n (n per patient)							

†STarT MSK scored 0-4, low risk; 5-8 medium risk; 9-12 high risk.

Example indicators (as in pilot paper):-

Intervention: Reduced (<0.04) [illegible] Increase [illegible].04) Provided earl[illegible]

Table 5a: Summary of overall and monthly follow up NRS-Pain (primary outcome measure) scores and between-group differences

Month	n_{SC}, n_{UC}	SC mean (SD)	UC mean (SD)	mean difference# (95% CI)	SMD (95% CI)	P value
1						
2						
3						
4						
5						
6$						
Average†*^						

SC = Stratified Care; UC = Usual Care. n_{SC} = number of data analysed in the SC arm; n_{UC} = number of data analysed in the UC arm.
# Between-group difference in mean scores (Usual Care – Stratified Care) by linear mixed model with Practice and Participants (random factors) and practice size and participants' age, gender and (point-of-consultation) pain score (fixed factors)
$ If the last monthly SMS/brief questionnaire response is missing it will be imputed using the corresponding pain response from the returned 6-month questionnaire (if available and completed within 20 days of the date of issue of their 6-month SMS/brief questionnaire)
† Primary endpoint (average of available data for 1-6 months follow up).
SMD: Standardized mean difference (effect size) relative to overall baseline SD.
* CACE estimate: mean difference XX (95% CI XX, XX); SMD XX (95% CI XX, XX); P=XX.
^ Sensitivity analysis (adjusted for xx, xx, … practice- and/or participant- baseline variables that were significantly different in Table 1 and 2): mean difference XX (95% CI XX, XX); SMD XX (95% CI XX, XX); P=XX.
Possible MI sensitivity analysis if imbalanced follow up between arms and missingness pattern is MAR or NMAR: mean difference XX (95% CI XX, XX); SMD XX (95% CI XX, XX); P=XX.

Table 5b: Number (%) achieving the MCID for the NRS-Pain (1-point change) and between-group differences

Month	SC n (%)	UC n (%)	OR# (95% CI)	% difference (95% CI)##	NNT (95% CI)
1					
2					
3					
4					
5					
6$					
Average					

SC = Stratified Care; UC = Usual Care.
# Between-group comparison (ratio of Stratified Care to Usual Care) by generalised mixed model with Practice and Participants (random factors) and practice size and participants' age, gender and (point-of-consultation) pain score (fixed factors).
Derived from the odds ratio using the observed proportion in the UC arm as the reference.
NNT = Number Needed to Treat (derived as the reciprocal of the % difference)

Table 6a: Subgroup analysis of NRS-Pain: Descriptive summary mean (SD) scores by STarT MSK risk subgroups

	Low risk		Medium risk		High risk	
**Month**	SC	UC	SC	UC	SC	UC
Clinical version						
1						
2						
3						
4						
5						
6						
Average						
Self-report version						
1						
2						
3						
4						
5						
6						
Average						

SC = Stratified Care; UC = Usual Care. Average = mean of available 1-6 month follow up data.

Table 6b: Subgroup analysis of NRS-Pain: Descriptive summary mean (SD) scores by pain region

	Back		Neck		Shoulder		Knee		Multi-Site	
**Month**	SC	UC	SC	UC	SC	UC	SC	UC	SC	UC
1										
2										
3										
4										
5										
6										
Average										

SC = Stratified Care; UC = Usual Care. Average = mean of available 1-6 month follow up data.

Table 7: Summary of overall and monthly NRS-psychological distress and NRS-pain self-efficacy (secondary outcome measures)

Month	n_{SC}, n_{UC}	SC mean (SD)	UC mean (SD)	mean difference# (95% CI)	SMD (95% CI)	P value
Psychological distress						
1						
2						
3						
4						
5						
6$						
Average#						
Pain self-efficacy						
1						
2						
3						
4						
5						
6$						
Average#						

SC = Stratified Care; UC = Usual Care. n_{SC} = number of data analysed in the SC arm; n_{UC} = number of data analysed in the UC arm.

Between-group difference in mean scores (Usual Care – Stratified Care) by linear mixed model with Practice and Participants (random factors) and practice size and participants' age, gender and (point-of-consultation) pain score and corresponding baseline questionnaire psychological distress or pain self-efficacy score (fixed factors). Average = mean of available 1-6 month follow up data.

$ If the last monthly SMS/brief questionnaire response is missing it will be imputed using the corresponding psychological distress / pain self-efficacy response from the returned 6-month questionnaire (if available and completed within 20 days of the date of issue of their 6-month SMS/brief questionnaire)

Table 8: Summary of secondary health outcomes from the 6 month follow up questionnaire

Key characteristics	n_{SC}, n_{UC}	SC	UC	Mean difference[1] or OR[2] (95% CI)##	P-value
Pain intensity (0-10 NRS)#, mean (SD)					
Overall pain change (-5 to +5)#, mean (SD)					
Distress (0-10 NRS)#, mean (SD)					
Confidence to manage (0-10 NRS)#, mean (SD)					
Days of moderate activity in last week, mean (SD)					
Physical Function, mean (SD)					
Back (RMDQ)					
Neck (NDI)					
Shoulder (SPADI-Function subscale)					
Knee (KOOS-PS)					
Multi-site (SF12 PCS)					
Standardised function score (0, 1)					
MSK-HQ (0-56)#, mean (SD)					
STarT MSK tool (Clinical version), mean (SD)					
STarT MSK (Clinical version) - risk subgroup, n (%)					
Low risk (0-4 score)					
Medium risk (5-8 score)					
High risk (9-12 score)					
STarT MSK tool (Self-report version), mean (SD)					
STarT MSK (Self-report version) - risk subgroup, n (%)					
Low risk (0-4 score)					
Medium risk (5-8 score)					
High risk (9-12 score)					
Health-related Quality of Life (EQ-5D), mean (SD)					
Fear Avoidance behaviour (TSK-11), mean (SD)					
Currently employed, n (%)					
No					
Yes					
Non-manual, n (% of Yes)					
Manual					
Performance at work over last 6 months (0-10 NRS)#, mean (SD)					
Time off work in last 6 months, n (%)					
No					
Yes					
No. of days absent (of Yes subgroup), mean (SD)					
Satisfaction with care, n (%)					
Very satisfied					
Quite satisfied					
No opinion					
Not very satisfied					

Not at all satisfied

SC = Stratified Care; UC = Usual Care. n_{SC} = number of data analysed in the SC arm; n_{UC} = number of data analysed in the UC arm.
# Performance at work (0-10 NRS) - where 0=problem 'not at all' affected performance over last 6 months, 10='so bad I am unable to do my job'; NRS-Pain 0='no pain', 10='worst ever pain'; NRS-Distress 0='no distress', 10='extreme distress'; NRS-Confidence to manage 0='not at all confident', 10='extremely confident'; MSK-HQ = Musculoskeletal Health Questionnaire (0-56 scale) based on summation of 14-items on a 0-4 scale and where 0=worst musculoskeletal health status and 56=best musculoskeletal health status; Pain change scale 11-point NRS scale (-5 to +5) where -5=very much worse, 0=unchanged, +5=completely recovered [change from clinic appointment to time of self-report baseline completion]; RMDQ = Roland & Morris Disability Questionnaire (0-24 scale) where 0=no low back pain/disability, 24=maximum low back/pain disability; NDI = Neck Disability Index (0-50 scale) where 0=no disability, 50=maximum disability; SPADI = Shoulder Pain & Disability Index (0-100) 0=no disability, 100=maximum disability); KOOS-PS = Knee Injury and Osteoarthritis Outcome Score Physical Function Short-form (0-100) 0=extreme disability, 100=no disability; SF12-PCS = Short Form 12v2 Physical Component Scale (0-100) 0=worst physical health score, 100=best physical health score. ECRQ …; TSK-11 = Tampa Scale of Kinesiophobia (11-44) 11=minimum fear avoidance, 44=maximum fear avoidance; STarT MSK Tool score (0-12) 0=lowest risk, 12=highest risk.
Between-group difference in mean scores (Usual Care – Stratified Care) and odds ratio (Usual Care / Stratified Care) respectively by linear and generalised mixed models with Practice (random factor) and practice size and participants' age, gender and (point-of-consultation) pain score and corresponding baseline measure response if available (fixed factors).

Table 9: Unit cost values for health care resources

Resource	Unit cost	Source
General practitioner		
Primary care nurse		
Consultant / Specialist / Hospital doctor		
Physiotherapist		
Acupuncturist		
Osteopath / Chiropractor		
Investigation resources	Variable	
Inpatient stay days – per day		
Medication (Prescribed)	Variable	

Sources: British National Formulary (BNF), Unit Costs of Health and Social Care and NHS Reference costs (BNF 2017; DoH 2017 ; Curtis and Burns, 2017).

Table 10: Summary of resource use and costs by study arm

	SC		UC	
	Mean per participant	Mean (SD) cost (£) per participant	Mean per participant	Mean (SD) cost (£) per participant
Health care professional visits				
General practitioner – General Practice				
- General Practice				
- NHS				
- Private				
Primary care nurse				
- General Practice				
- NHS				
- Private				
Consultant / Specialist / Hospital doctor				
- General Practice				
- NHS				
- Private				
Physiotherapist				
- General Practice				
- NHS				
- Private				
Acupuncturist				
- General Practice				
- NHS				
- Private				
Ostoepath / Chiropractor				
- General Practice				
- NHS				
- Private				
Other health care professionals				
- General Practice				
- NHS				
- Private				
Investigations				
X-rays				
- NHS				
- Private				
etc… (as listed by participants)				
Inpatient days for back pain				
- NHS				
- Private				
Prescriptions				

Data from 6 month self-report questionnaire (except prescription which are from medical record linkage)

Table 11: Summary of work-related outcomes for participants in paid employment at baseline, by intervention arm

	SC (n=XX)	UC (n=XX)	Mean difference# (95% CI)
Work absenteeism: Time off work in last 6 months, n (%)			
No			
Yes			
No. of days absent, mean (SD)			
Work presenteeism: Performance at work over last 6 months (0-10 NRS)*, mean (SD)			
Reduced productivity days, mean (SD)			
Indirect Cost (£) of work absenteeism, mean (SD)			
Indirect Cost (£) of work presenteeism, mean (SD)			

SC = Stratified Care; UC = Usual Care.
# UC minus SC
* Performance at work (0-10 NRS) - where 0=problem ‘not at all’ affected performance over last 6 months, 10=’so bad I am unable to do my job’

Table 12: Summary of aggregated costs (£) by intervention arm and between-group differences.

	SC	UC
Imputed analysis		
Total NHS cost (base-case)		
Mean difference (95% CI)		
Total Healthcare cost		
Mean difference (95% CI)		
Total Societal cost (excluding work presenteeism)		
Mean difference (95% CI)		
Total Societal cost (including work presenteeism)		
Mean difference (95% CI)		
Complete-case (sensitivity) analysis		
Total NHS cost (base-case)		
Mean difference (95% CI)		
Total Healthcare cost		
Mean difference (95% CI)		
Total Societal cost (excluding work presenteeism)		
Mean difference (95% CI)		
Total Societal cost (including work presenteeism)		
Mean difference (95% CI)		

SC = Stratified Care; UC = Usual Care.

Table 13: Descriptive and incremental health outcomes over 6 months for the base-case and complete-case analyses

	SC	UC	Mean difference# (95% CI)
*Primary (Imputed) analysis**			
Baseline EQ-5D			
Month 6 EQ-5D			
QALYs			
Adjusted QALYs*	-	-	
Complete-case analysis			
Baseline EQ-5D, n=XX			
Month 6 EQ-5D, n=XX			
QALYs, n=XX			
Adjusted QALYs*	-	-	

SC = Stratified Care; UC = Usual Care; QALY= Quality Adjusted Life Years. Values to denote mean (SD) unless otherwise stated. # Between-group difference in mean scores. * Incremental QALY estimates following multiple regression-based adjustment for age, gender and baseline EQ-5D.

Table 14: Subgroup analysis: Costs and health outcomes mean (SD) scores by STarT MSK risk subgroups (Imputed analysis)

	**Low risk**		**Medium risk**		**High risk**	
	SC	UC	SC	UC	SC	UC
Total NHS cost						
Mean difference (95% CI)						
Total Health care cost Mean						
difference (95% CI) Total						
Societal cost						
Mean difference (95% CI)						
Baseline EQ-5D						
Month 6 EQ-5D						
QALYs						
Mean difference (95% CI)						
Adjusted QALYs *						
Mean difference (95% CI)						

SC = Stratified Care; UC = Usual Care * Incremental QALY estimates following multiple regression-based adjustment for age, gender and baseline EQ-5D
